# The production of esters by specific sourdough lactic acid bacteria species is limited by the precursor concentrations

**DOI:** 10.1128/aem.02216-24

**Published:** 2025-02-27

**Authors:** Inés Pradal, Stefan Weckx, Luc De Vuyst

**Affiliations:** 1 Research Group of Industrial Microbiology and Food Biotechnology (IMDO), Department of Bioengineering Sciences, Faculty of Sciences and Bioengineering Sciences, Vrije Universiteit Brussel 70493 , Brussels, Belgium; Universita degli Studi di Napoli Federico II, Portici, Italy

**Keywords:** sourdough, lactic acid bacteria, yeasts, esters, fruity flavor, genome mining

## Abstract

The production of fruity esters by sourdough lactic acid bacteria (LAB) and yeasts has not been explored in detail. Moreover, the biosynthesis of esters by LAB species under conditions similar to those occurring during sourdough production is still questionable. Concerning yeasts, a genome mining of 75 genomes revealed a strain dependency of the presence of seven specific ester biosynthesis genes. Accordingly, PCR assays to detect these acetate (*ATF1* and *ATF2*) and ethyl ester (*EHT1* and *EEB1*) biosynthesis genes were developed and used to screen 91 strains of yeast species. Concerning LAB, a genome mining of 401 genomes revealed a species dependency of the presence of three esterase-encoding genes (*estA*, *estB*, and *estC*). A phenotypic analysis carried out with a selection of 10 strains of the LAB species *Companilactobacillus crustorum*, *Companilactobacillus nantensis*, *Companilactobacillus paralimentarius, Fructilactobacillus sanfranciscensis*, *Lactiplantibacillus xiangfangensis*, *Levilactobacillus zymae*, and *Limosilactobacillus fermentum* in a wheat sourdough simulation medium (WSSM) supplemented with ester precursor molecules ([higher] alcohols and fatty acids) revealed that their ester biosynthesis capacity was limited by the precursor concentrations. Ethyl acetate and ethyl lactate were produced by all strains, except for those of *Frul. sanfranciscensis*. These results suggested that one of the esterase-encoding genes considered could be implicated in the ethyl acetate and/or ethyl lactate biosynthesis. Overall, the ester biosynthesis capacity by LAB is of great interest in view of fruity flavor formation during sourdough and sourdough bread productions.

**IMPORTANCE:**

The present study gave insights into the production of esters, which impart fruity flavors to fermented foods, by not only sourdough yeasts but also lactic acid bacteria. It showed that some lactic acid bacteria species can synthesize the esters ethyl acetate (sweet notes) and ethyl lactate (creamy notes) under specific conditions. The information gathered during the present study will enable sourdough bakers and companies from the bakery sector to get more information on how to produce sourdoughs that can add fruity notes to the final products after a rational screening and selection of potential starter culture strains.

## INTRODUCTION

The introduction of baker’s yeast and the application of short bread dough fermentation steps in bakery practices has led to less flavorful bread crumbs (1). However, the taste and aroma of breads are some of the main characteristics considered by consumers to determine the quality of baked goods (2, 3). Therefore, in the past decades, the ancient use of sourdough for bread production has been on the rise again, as sourdoughs are a source of flavor (3–5). Sourdoughs contribute not only sourness but also freshness, fruitiness, and roastiness to breads (6). Indeed, comparisons between chemically acidified breads and sourdough breads have shown that the latter possess a higher sensory quality (3).

The presence of flavor compounds in sourdough-containing baked products can be the result of the fermentation process, Maillard reactions, or lipid oxidation (3, 4, 7). The crumb flavor is mainly the result of the fermentation steps at the sourdough and bread dough preparation levels (3, 7), the fermentation step during the bread making process having a great impact (4, 7, 8). Hence, different flavor profiles resulting from a diverse sourdough microbiota determine differences in the sensory characteristics of the crumbs of sourdough-containing breads (9–11) and steamed breads (12, 13). To enrich the sourdough matrix with desirable flavor compounds, studying how these compounds are produced by the different sourdough microorganisms is thus of crucial importance, in particular, to be able to rationally select starter culture strains for sourdough production.

Among the volatile organic compounds (VOCs) produced by sourdough microorganisms, esters are of particular interest because of their fruity notes. Esters typically characterize the flavor of not only several beers, wines, and other alcoholic beverages (14–22) but also cocoa (23, 24), cheese (25–27), and sourdough (28–30). Fruity esters are the result of a condensation reaction between an acyl-CoA moiety and an alcohol moiety (31, 32), yielding either acetate esters (with acetyl-CoA as acyl-CoA moiety) or ethyl esters (with ethanol as alcohol moiety) (33, 34). They are mainly produced by yeasts (17, 31, 34). In yeasts, the biosynthesis of acetate esters is catalyzed by acetyl-CoA transferases, which condense acetyl-CoA with a higher alcohol. The *ATF1* and *ATF2* (alcohol acetyl transferase 1 and 2, respectively) genes that encode such acetyl-CoA transferases have been described in *Saccharomyces cerevisiae*, and the corresponding enzymes have been linked to the production of isoamyl acetate (banana notes) and ethyl acetate (sweet notes) (35–40). The ethyl ester biosynthesis by yeasts is catalyzed by acyl-CoA transferases or esterases (17, 34). The genes *EHT1* (ethyl hexanoyl transferase), *EEB1* (ethyl ester biosynthesis), and *YMR210w* (a member of the *EHT1* and *EEB1* gene clade) have been described in *S. cerevisiae* (41, 42). The enzyme EHT1 has been linked to the production of ethyl hexanoate (apple notes) and ethyl octanoate (apricot notes), EEB1 to that of ethyl butanoate (pineapple notes), ethyl hexanoate, ethyl octanoate, and ethyl decanoate (grape notes), and the protein of *YMR210w* to that of ethyl octanoate and ethyl decanoate. In addition, two acyl-CoA transferases encoded by the genes *EAT* (ethanol acetyltransferase) and *IMO32* (an *EAT* homolog) participate in ethyl acetate biosynthesis by *S. cerevisiae* and *Wickerhamomyces anomalus* (43, 44).

In contrast, ester production by LAB under conditions similar to those that occurred during sourdough production is still questionable. LAB possess lipases as well as esterases, which are enzymes capable of hydrolyzing and synthesizing ester bonds (26). The former ones are active at the water-lipid interphase in oil-in-water emulsions to hydrolyze water-insoluble substrates, whereas the latter ones act only in aqueous solutions and prefer water-soluble substrates. Ester biosynthesis during sourdough and bread production should take place in the aqueous phase and via esterification of free acyl-CoA and alcohol moieties, and, therefore, it should be catalyzed by esterases. This ester biosynthesis capacity has, under comparable conditions, only been tested in a few cases, in particular with the enzymes EstA, EstB, and EstC, which were discovered as ester hydrolases in LAB species (45–47) but that catalyze the biosynthesis of ethyl butanoate, ethyl pentanoate (apple and pineapple notes), and ethyl hexanoate from the free moieties (48). Also, several genes and enzymes from sourdough-related LAB species have been characterized, such as lipases and esterases from *Lacticaseibacillus casei* (46), *Lactiplantibacillus plantarum* (49–54), *Lactobacillus helveticus* (45), *Lentilactobacillus hilgardii* (55), *Lactococcus lactis* (56, 57), and *Limosilactobacillus fermentum* (58), but they have not been tested under comparable conditions. Studies on ester production by LAB mainly deal with the detection of esters in fermented food products made with LAB and their *in vitro* biosynthesis. Concerning the latter, experimental assays are generally limited to the degradation of *p*-nitrophenyl esters, β-naphthyl esters, or the synthetic glyceride substrate tributyrin (25, 26, 45–47, 49–52, 55–59). Only few studies have reported assays carried out with the free precursor molecules but without a link to specific enzymatic activities (60, 61). Nevertheless, different ester profiles as a result of the use of specific LAB starter cultures have been described for cheese (62), shalgam (63), wine (64, 65), sourdough (66–70), and sourdough-containing bread (11, 71). However, in most of these fermented foods, yeasts are part of the microbiota and, consequently, ascribing the ester biosynthesis activity to LAB is not obvious. Yet, different ester profiles can be identified during sourdough production when different LAB strains are inoculated and yeasts are inhibited by sterilization of the flour or the addition of antibiotics (66, 68).

The aim of the present study was to unravel the ester biosynthesis potential of selected sourdough yeast and LAB strains by means of an *in silico* genome mining analysis, followed by PCR assays for genes involved in ester biosynthesis in the case of the yeast strains and a phenotypic screening, encompassing fermentation processes in a wheat sourdough simulation medium (WSSM) for ester production capacity in the case of the LAB strains.

## RESULTS

### Genome retrieval and phylogenetic tree construction

#### Yeasts

A literature search for sourdough yeasts yielded at least 39 different species that occur in spontaneous backslopped sourdoughs. Among those, six species had no genome sequence available in the Genome database of the National Center for Biotechnology Information (NCBI; https://www.ncbi.nlm.nih.gov/genome). For the other 33 yeast species, a total of 75 genomes of concomitant strains were retrieved. Among those, 34 genomes were complete (complete assembly level) and five genomes were not complete (scaffold level), but they were from strains derived from sourdoughs (Table S1). Furthermore, 14 genomes were from strains derived from other food products.

#### Lactic acid bacteria

A literature search for sourdough LAB yielded at least 81 different species that occur in spontaneous backslopped sourdoughs. A total of 401 genomes were retrieved from 68 LAB species that belonged to the former *Lactobacillus* genus (Table S2). Among those, 340 genomes (85%) were complete (complete assembly level). For only 11% of the 401 LAB genomes, the source of the concomitant strains was unknown; 232 LAB genomes (57%) were from strains derived from food products, including 61 from sourdoughs.

### *In silico* genome mining of ester biosynthesis genes

#### Yeasts

**Fig 1:**
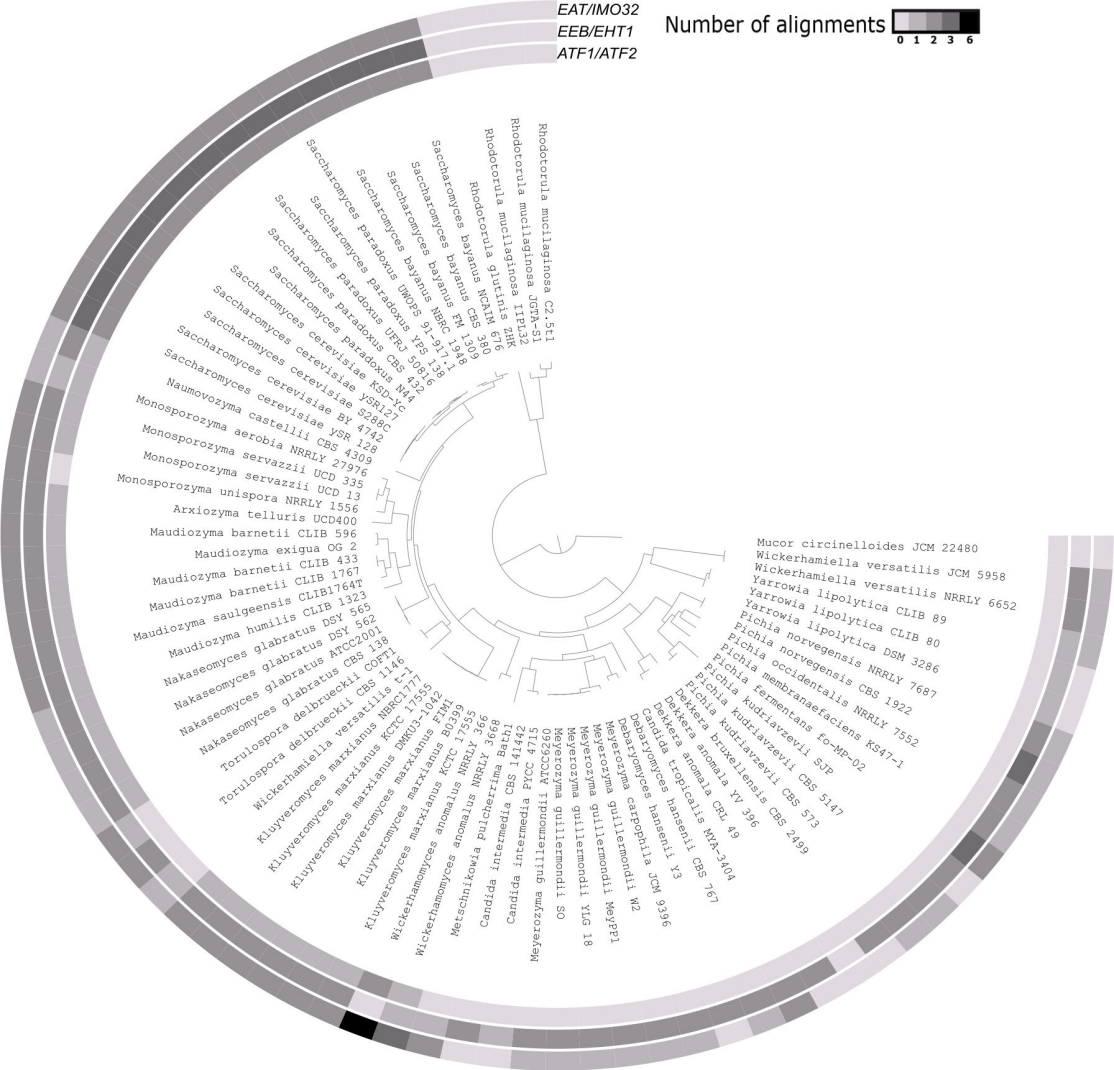
Phylogenetic tree based on amino acid sequences derived from the yeast genomes used for a genome mining analysis targeting ester biosynthesis genes, and the number of acetate ester (*ATF1*/*ATF2*), ethyl ester (*EEB*/*EHT1*), or ethyl acetate (*EAT*/*IMO32*) biosynthesis genes present in each genome (varying shading intensity).

Genome mining showed that the amino acid sequences representing the three different types of ester-producing enzymes, corresponding with the acetate ester biosynthesis genes *ATF1* and *ATF2*, the ethyl ester biosynthesis genes *EEB1*, *EHT1*, and *YMR210w*, and the ethyl acetate biosynthesis genes *EAT* and *IMO32*, aligned per type to the same genomic region. Indeed, as these amino acid sequences shared >30% sequence identity, it was not possible to assign the different regions to the appropriate enzyme-encoding genes. Therefore, a manual curation of the alignments was carried out to avoid an overestimation of the number of genes present in each yeast genome. This showed that the occurrence of genes involved in acetate ester, ethyl ester, and ethyl acetate biosynthesis was strain dependent (Fig. 1).

A maximum of eight genes in a single yeast genome were detected in the genome of *W. anomalus* NRRLY 0366 (Fig. 1). The maximum numbers of genes for each of the ester types mentioned above were two, three, and six, respectively, which was the case for the genomes of strains of *W. anomalus*, *Saccharomyces bayanus*, *Saccharomyces paradoxus*, and *S. cerevisiae*. None of the genes responsible for the biosynthesis of acetate esters, ethyl esters, and ethyl acetate were found in 38, 8, and 14 of the yeast genomes examined, respectively. Moreover, a total of 25 yeast genomes did not harbor any of these genes. It concerned genomes from strains of the yeast species *Dekkera anomala*, *Rhodotorula circinelloides, Rhodotorula glutinis,* and *Rhodotorula mucilaginosa*. None of the yeast genomes examined contained acetate ester biosynthesis genes only, whereas ethyl ester or ethyl acetate biosynthesis genes were solely present in eight and two yeast genomes, respectively. Moreover, 23 yeast genomes encompassed genes related to both ethyl ester and ethyl acetate biosynthesis.

#### Lactic acid bacteria

**Fig 2:**
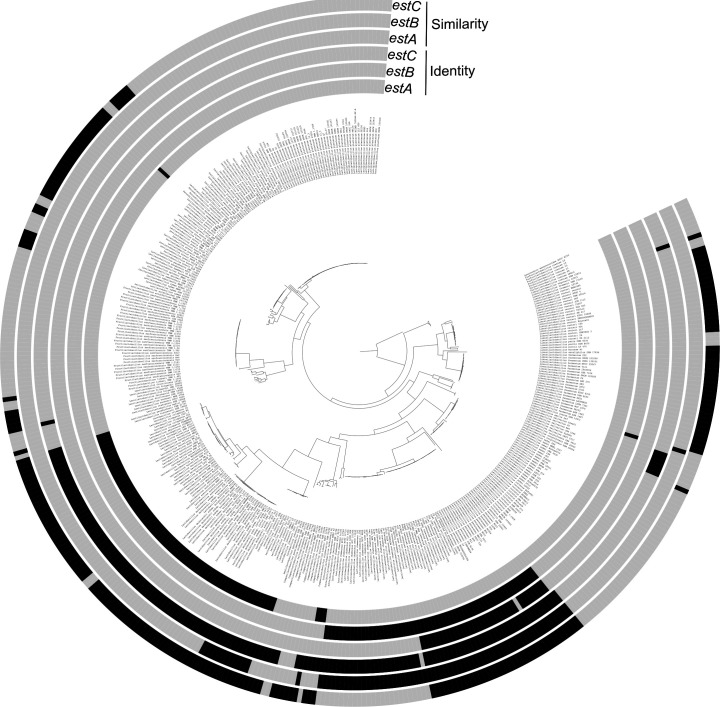
Phylogenetic tree based on gene sequences of the lactic acid bacteria (LAB) genomes used for a genome mining analysis targeting three esterase genes, and the presence (black) or absence (gray) of these genes, considering hits with >80% query coverage and >30% sequence identity (identity) or >80% query coverage and >30% sequence similarity (similarity).

In contrast to the yeast genomes, the presence of the ester biosynthesis genes *estA, estB,* and *estC*, encoding the concomitant esterases, in the LAB genomes was species dependent, as all genomes of strains of the same LAB species showed the same number of hits for each gene, except for the genomes from strains of *Lacticaseibacillus paracasei* (Fig. 2; Table S2). All LAB genomes considered harbored only one copy of the same gene. Moreover, the three genes assessed were not widely distributed in the LAB genomes, as the genomes of 49 of the 68 LAB species examined did not harbor any of the genes solely. The *estA* and *estB* genes were present in the genomes of 13 and 3 of the 68 LAB species examined, respectively. The *estC* gene as the sole ester biosynthesis gene was not present in any of the LAB genomes considered, but it was present together with the *estB* gene in the genomes of *Lacc. paracasei*.

A less stringent analysis showed that the genomes of 24 of the 68 LAB species examined did not harbor any copy of the genes targeted (Fig. 2; Table S2). The *estA* and *estB* genes were present in the genomes of three and two of the 68 LAB species examined, respectively. The *estC* gene was the most widely distributed gene, being present in the genomes of 17 of the 68 LAB species examined. The *estA* and *estB* genes were both present in all genomes of *Lacc. rhamnosus*. The *estA* and *estC* genes were both present in the genomes of 16 of the 68 LAB species examined.

In the case of the genomes of sourdough LAB strains, at least one of the genes was present only in the genomes of strains of *Companilactobacillus crustorum, Companilactobacillus mindensis, Companilactobacillus paralimentarius, Levilactobacillus hammesii, Levilactobacillus zymae,* and *Liml. fermentum*. None of the LAB genomes considered harbored all three genes, whereas the *estA* and *estC* genes were present in the genomes of strains of *Coml. nantensis* and *Coml. paralimentarius*. None of the *Fructilactobacillus sanfranciscensis* genomes possessed any of the three genes targeted.

None of the regions found were annotated as EstA, EstB, or EstC. Nevertheless, all regions that aligned with the *estB* or *estC* genes and 70% of those that aligned with the *estA* gene were annotated as α/β-hydrolases.

### PCR assays for the detection of ester biosynthesis genes in yeast genomic DNA

#### Optimization of the PCR assays

Two PCR assays were successfully developed, one for the detection of acetate ester biosynthesis genes (*ATF1*/*ATF2*) and another for ethyl ester biosynthesis genes (*EEB1*/*EHT1*) present in yeast genomes. After the optimization of the PCR conditions, the PCR assays to amplify a fragment of the concomitant genes considered resulted in an amplicon of 300 bp and 500 bp, respectively.

#### PCR screening of sourdough yeast isolates

Genomic DNA of 91 yeast strains with a sourdough origin (Table S3) was subjected to the optimized PCR assays mentioned above. Twenty-six yeast strains harbored both types of ester biosynthesis genes, encompassing 25 *S*. *cerevisiae* strains and the *Maudiozyma bulderi* E-B-W-Y24 strain. Only 10 yeast strains had the acetate ester biosynthesis genes, whereas 29 yeast strains possessed the ethyl ester biosynthesis genes. Finally, 26 yeast strains did not harbor any of the ester biosynthesis genes targeted.

### LAB genome sequencing and *in silico* genome mining for the detection of ester biosynthesis genes

The genome sequences of 14 selected LAB strains isolated from sourdough (Table 1) were successfully obtained by means of Oxford Nanopore Technology (ONT) and Illumina sequencing. All strains, except for *Coml. paralimentarius* R19081, *Coml. paralimentarius* BBRM18, and *Levl. zymae* LMG 22198, possessed at least one plasmid. Subsequently, these genomes, together with the genomes of *Liml. fermentum* IMDO 130101 (72) and *Coml. crustorum* LMG 23699 (73), were subjected to genome mining for the detection of the ester biosynthesis genes *estA*, *estB*, and/or *estC*. The *estA* gene was present in the genome of the *Coml. nantensis* strain and in the genomes of all strains of *Coml. paralimentarius*, whereas the *estB* gene was only present in the genome of one strain of *Coml. paralimentarius* (EBRM1). The *estC* gene occurred in all LAB genomes examined.

**TABLE 1:** Lactic acid bacteria strains used in the present study for genome sequencing and genome mining to target three esterase genes (G) and/or to perform a phenotypic screening as to their ester biosynthesis capacity (P)^*b*^

Strain	Isolation source	Genome accession number	Genome length (Mbp)	Genome coverage	Assembly level	Analysis performed
*Companilactobacillus crustorum* LMG 23699	Belgian bakery sourdough (74)	GCF_001438825.1 ^*a*^	2.24	100	87 scaffolds	P
*Companilactobacillus crustoru*m IMDO BLIM15	Belgian laboratory sourdough (IMDO-VUB)	ERS16236860	2.31	110	1 circular chromosome + 2 circular plasmids	G
*Companilactobacillus crustorum* IMDO BLWM12	Belgian laboratory sourdough (IMDO-VUB)	ERS16236861	2.31	467	1 circular chromosome in 7 contigs + 3 circular plasmids	G
*Companilactobacillus crustorum* IMDO 1Mg81	Belgian laboratory sourdough (IMDO-VUB)	ERS16236864	2.72	227	1 circular chromosome + 1 circular plasmid	G
*Companilactobacillus nantensis* R19088	Belgian bakery sourdough (IMDO-VUB)	ERS16236870	2.73	554	123 chromosomal contigs + 6 plasmid contigs	G+P
*Companilactobacillus paralimentarius* IMDO 1Mg86	Belgian laboratory sourdough (IMDO-VUB)	ERS16236865	2.73	92	1 linear chromosome + 2 circular plasmids	G+P
*Companilactobacillus paralimentarius* IMDO 1Mg105	Belgian laboratory sourdough (IMDO-VUB)	ERS16236863	2.73	302	1 circular chromosome + 1 circular plasmid	G+P
*Companilactobacillus paralimentarius* IMDO BBRM18	Belgian household sourdough (75)	ERS16236866	2.76	200	1 circular chromosome	G+P
*Companilactobacillus paralimentarius* IMDO EBRM1	Belgian bakery sourdough (75)	ERS16236867	2.65	99	1 circular chromosome + 1 circular plasmid	G+P
*Companilactobacillus paralimentarius* R18618	Belgian bakery sourdough (IMDO-VUB)	ERS16236872	2.81	60	1 circular chromosome + 2 circular plasmids	G
*Companilactobacillus paralimentarius* R19079	Belgian bakery sourdough (IMDO-VUB)	ERS16236868	2.75	498	1 circular chromosome + 3 circular plasmids	G
*Companilactobacillus paralimentarius* R19081	Belgian bakery sourdough (IMDO-VUB)	ERS16236869	2.81	175	1 circular chromosome + 2 circular plasmids	G
*Companilactobacillus paralimentarius* R19092	Belgian bakery sourdough (IMDO-VUB)	ERS16236871	2.65	343	1 circular chromosome + 2 circular plasmids	G
*Fructilactobacillus sanfranciscensis* ACA-DC 3378	Greek household sourdough (76)	ERS16380042	1.37	192	1 circular chromosome + 1 circular plasmid	P
*Fructilactobacillus sanfranciscensis* LMG 16002^T^	San Francisco sourdough (76)	–	–	–	–	P
*Lactiplantibacillus xiangfangensis* IMDO EBRMM8	Belgian bakery sourdough (75)	ERS16236876	3.06	54	1 circular chromosome + 2 circular plasmids	G
*Levilactobacillus zymae* LMG 22198^T^	Wheat sourdough (76)	ERS16236877	2.78	53	1 circular chromosome	G+P
*Limosilactobacillus fermentum* IMDO 130101	Belgian bakery sourdough (77)	GCF_900205745.1 ^*a*^	2.10	37	1 circular chromosome	P

^*a*^ Genomes available in the National Center for Biotechnology Information (NCBI) Genome database (https://www.ncbi.nlm.nih.gov/genome) at the time of analysis. All genomes starting with ERZ are available in the European Nucleotide Archive (ENA; https://www.ebi.ac.uk/ena/browser/home). The genome coverage of these genomes sequenced during the present study is that obtained by long-read sequencing (Oxford Nanopore Technology).

^*b*^ IMDO-VUB, research group of Industrial Microbiology and Food Biotechnology.

### Phenotypic screening of LAB strains

#### Microbial growth in wheat sourdough simulation medium (WSSM) and modified WSSM (mWSSM)

The 10 LAB strains examined, namely, *Coml. crustorum* LMG 23699, *Coml. nantensis* R19088, *Coml. paralimentarius* 1Mg86, 1Mg105, BBRM18, and EBRM1, *Frul. sanfranciscensis* ACA-DC 3378 and LMG 16002^T^, *Levl. zymae* LMG 22198, and *Liml. fermentum* IMDO 130101, grew in both WSSM and mWSSM. However, the lag phase of all strains of *Coml. crustorum*, *Coml. nantensis*, *Coml. paralimentarius*, and *Liml. fermentum* tested was longer, and the maximum population density as well as the specific growth rate was lower in mWSSM than in WSSM (Fig. S1). On average, the maximum population density and the specific growth rate of the strains grown in mWSSM were 67% and 68% of the values obtained in WSSM, respectively. Moreover, as a consequence of a lower specific growth rate, the exponential growth phase was longer in mWSSM than in WSSM. In contrast, the growth of strains of *Frul. sanfranciscensis* and *Levl. zymae* showed a similar duration of the lag and exponential growth phases in both media.

#### Ester biosynthesis by LAB strains

Whereas the pH of the non-inoculated media was stable during 28 h in both cases, i*.*e., 6.6 and 4.8 in WSSM and mWSSM, respectively (Fig. S2), this value decreased with the fermentation time when these media were inoculated with each of the 10 LAB strains examined. A similar end-value was reached in both media for each of the strains of *Coml. crustorum*, *Coml. nantensis*, and *Coml. paralimentarius* tested. However, in the case of the *Frul. sanfranciscensis* strains, *Levl. zymae* strain, and *Liml. fermentum* strain, the end-pH was slightly higher in WSSM than in mWSSM.

**Fig 3:**
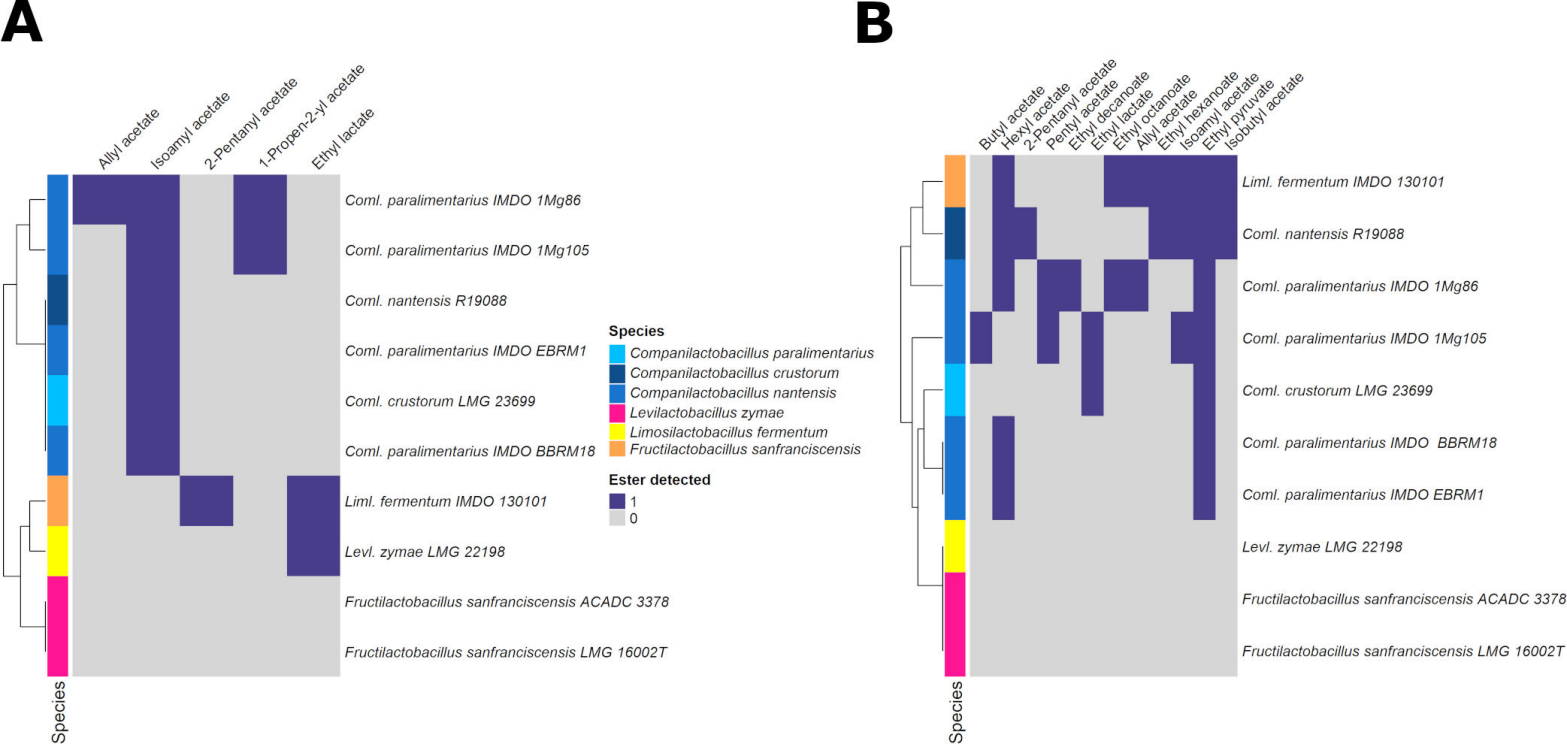
Heatmap showing the biosynthesis of esters during fermentation processes carried out with a selection of lactic acid bacteria strains in a wheat sourdough simulation medium (**A**) and in a modified version of this medium containing the ester precursor molecules (**B**).

Among the VOCs detected by headspace/solid-phase microextraction coupled to gas chromatography with time-of-flight mass spectrometry (HS/SPME-GC-TOF-MS), taken all results together, seven different esters were found during the fermentation processes carried out with all LAB strains tested in WSSM, namely, allyl acetate, ethyl lactate, ethyl acetate, ethenyl acetate, isoamyl acetate, 2-pentyl acetate, and 2-propen-2-yl acetate (Fig. 3A). However, the ethyl acetate and ethenyl acetate esters were detected with a higher z-score in uninoculated WSSM samples compared with most of the other samples, suggesting that these esters were produced by chemical reactions and that their presence could not be attributed to the growth of specific LAB strains. Ester biosynthesis occurred mainly at the end of the exponential growth phase and during the stationary phase. The *Coml. paralimentarius* 1Mg86 and 1Mg105 strains produced a maximum number of esters (three), namely, allyl acetate, isoamyl acetate, and 1-propen-2-yl acetate, whereas none of the esters mentioned above were found in cultures of any of the two strains of *Frul. sanfranciscensis* tested.

In the case of mWSSM, 13 esters were found when taking the results of all LAB strains tested together, more specifically, allyl acetate, butyl acetate, ethyl acetate, ethyl decanoate, ethyl hexanoate, ethyl lactate, ethyl octanoate, ethyl pyruvate, hexyl acetate, isoamyl acetate, isobutyl acetate, pentyl acetate, and 1-propen-2-yl acetate (Fig. 3B). However, the ethyl acetate ester was detected with a higher z-score in uninoculated mWSSM samples compared with most of the other samples. Again, ester biosynthesis mainly occurred at the end of the exponential growth phase and during the stationary phase. The strain of *Liml. fermentum* produced a higher number of esters (seven, namely, allyl acetate, hexyl acetate, ethyl hexanoate, ethyl octanoate, ethyl pyruvate, isoamyl acetate, and isobutyl acetate), followed by the strains *Coml. nantensis* R19088 (six, namely, hexyl acetate, 2-pentyl acetate, ethyl hexanoate, ethyl pyruvate, isoamyl acetate, and isobutyl acetate) and *Coml. paralimentarius* IMDO 1Mg86 (six, namely, allyl acetate, hexyl acetate, pentyl acetate, ethyl decanoate, ethyl octanoate, and ethyl pyruvate). In contrast, neither of the two strains of *Frul. sanfranciscensis* tested nor the *Levl. zymae* LMG 22198 strain produced any ester. The production of different esters by *Coml. paralimentarius* was strain dependent, as different strains of this species produced different esters in the media tested. All strains produced more esters during the mWSSM fermentation processes than during the WSSM fermentation processes.

**Fig 4:**
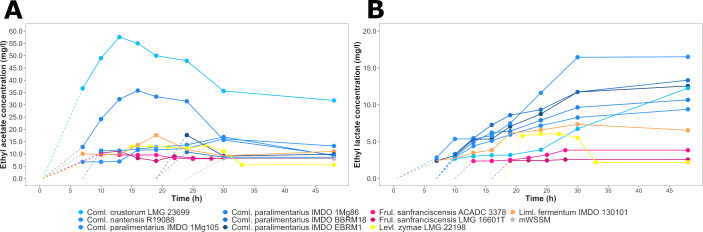
Production dynamics of the esters ethyl acetate (**A**) and ethyl lactate (**B**) during fermentation processes carried out with a selection of lactic acid bacteria strains in a modified version of wheat sourdough simulation medium (mWWSM) containing ester precursor molecules. The control fermentation processes were carried out in mWSSM without inoculation of any of the lactic acid bacteria strains. The average of two biological replicates is shown.

Among the esters targeted by means of liquid injection gas chromatography with triple-quadrupole tandem mass spectrometry (LI-GC-TQ-MS), none were produced above the quantification limit during the WSSM fermentation processes. Nevertheless, the ethyl acetate and ethyl lactate esters were produced during the mWSSM fermentation processes in concentrations higher than the quantification limit. The production of ethyl acetate differed among the LAB species and strains examined (Fig. 4A). During uninoculated fermentation processes and mWSSM fermentation processes carried out with the *Frul. sanfranciscensis* strains, ethyl acetate was produced in concentrations of, on average, 9.8 ± 1.3 mg/L after 24 and 18 h, respectively. As these concentrations were reached faster in inoculated fermentation processes than in uninoculated ones, it was likely that the production of ethanol and acetic acid by these LAB strains eased the ester formation by a chemical reaction. At the end of the mWSSM fermentation processes (48 h) carried out with strains of *Companilactobacillus* species and *Liml. fermentum* IMDO 130101, an average of 38.4 ± 8.4 mg/L of ethyl acetate was produced. However, the production dynamics of each strain were different. In particular, the *Coml. paralimentarius* IMDO 1Mg86 and *Coml. crustorum* LMG 23699 strains produced a maximum ethyl acetate concentration of, on average, 35.8 mg/L in mWSSM after 16 h of fermentation and 57.6 mg/L in mWSSM after 13 h of fermentation, respectively. The *Levl. zymae* LMG 22198 strain produced, on average, 13.5 mg/L of ethyl acetate in mWSSM after 27 h of fermentation. The concentrations of ethyl acetate decreased at the end of all mWSSM fermentation processes carried out.

The production of ethyl lactate differed among the species and strains tested (Fig. 4B). In uninoculated mWSSM fermentation processes, as well as in mWSSM fermentation processes carried out with *Frul. sanfranciscensis*, ethyl lactate was present at concentrations of, on average, 2.9 ± 0.7 mg/L. However, this concentration was reached faster in inoculated fermentation processes than in uninoculated ones, indicating that the production of ethanol and lactic acid by these LAB strains eased the ester formation by a chemical reaction. Strains from *Companilactobacillus* species produced, on average, 11.6 ± 1.4 mg/L of ethyl lactate in mWSSM, whereas *Liml. fermentum* IMDO 130101 and *Levl. zymae* LMG 22198 produced, on average, 7.4 and 6.1 mg/L of this ester, respectively.

## DISCUSSION

Whereas ester biosynthesis by yeasts is well known, delivering fruity flavors in many fermented foods and beverages produced with a fermentation microbiota involving yeasts (14, 16, 17, 20), it has only been shown sporadically for LAB (66, 68). The present study evaluated the ester biosynthesis capacity of sourdough yeast strains as well as sourdough LAB strains. For sourdough yeasts, the occurrence of genes encoding ac(et)yl-CoA transferases, enzymes involved in acetate ester, ethyl ester, or ethyl acetate biosynthesis (35–44), was strain dependent. Therefore, the PCR assays developed in the present study, targeting either acetate ester or ethyl ester biosynthesis genes, are a suitable tool to screen yeast strains as to their fermented food production potential based on their ester biosynthesis capacity. This strain dependency is also reflected in the esters constituting the flavor profile of, for instance, beers (18, 21, 22), wines (15, 19, 32), and even sourdoughs (4, 78–81). For sourdough LAB, the occurrence of the *estA*, *estB*, and *estC* genes, which encode ethyl ester biosynthesis enzymes (45–47), was species dependent. Whereas yeasts recruit the necessary ester precursor molecules from their carbohydrate, lipid, and amino acid metabolism (20), the ester formation by LAB seemed to be limited by the concentrations of the precursors that have to be derived from these metabolisms.

The *in silico* analysis carried out during the present study revealed the distribution of genes encoding enzymes involved in ester biosynthesis among several sourdough microorganisms, both yeasts and LAB. This *in silico* analysis was limited to those species whose genome was available in the NCBI Genome database. Given the strain dependency of the presence of ester biosynthesis genes in yeast genomes, the PCR assays developed during the present study allowed to screen and select yeast strains for the production of sourdoughs with an enhanced ester flavor profile. It needs to be mentioned that a positive result of the PCR assay did not necessarily mean that the strain harboring a gene of interest effectively produced the ester of interest, as there could be hurdles at the level of gene expression, mRNA translation, and enzyme efficiency. Conversely, a negative result of the PCR assay did not necessarily mean that the strain screened lacked a gene involved in ester biosynthesis, as the particular gene sequence might be deviating from the sequences that were used in the multiple sequence alignment (MSA) as part of the primer design procedure in such a way that the primers would not anneal. In addition, genes that have not been characterized so far but might be implicated in ester formation by sourdough yeast strains could not be targeted (17, 20, 31, 34). Yet, the necessary precursors should be produced to enable ester biosynthesis and, therefore, ester-producing yeast strains should possess and express the concomitant genes (31). Moreover, ester-degrading enzymes, such as Iah1 (an esterase), can be encoded by yeast genomes and, hence, affect the final ester concentration (31). Finally, the desirable ester production can also be influenced by the fermentation conditions that affect both yeast growth and concomitant gene expression and regulation (31, 82), such as the available carbohydrates (83, 84), available nitrogen (85–87), oxygen levels (39), and fermentation temperature (22, 88). Consequently, in view of enhanced ester formation during sourdough production, not only the right yeast strains should be selected, but also optimal fermentation conditions should be taken into account. Moreover, the yeast concentration (88) and the combination of different yeast strains could influence the final ester profile (18, 89–94).

Although the presence of the three esterases involved in ester biosynthesis in the LAB strains screened during the present study was species dependent, the presence of an esterase gene in wine lactobacilli that have been screened through PCR amplification is strain dependent (95). However, neither genetic information nor the phenotypic capability of ester biosynthesis was discussed in the latter study. The present study did show ester biosynthesis by sourdough LAB strains in an adapted fermentation medium, namely, mWSSM, containing the necessary water-soluble precursors, i*.*e., (higher) alcohols and fatty acids, through their detection by means of HS/SPME-GC-TOF-MS (presence/absence) and LI-GC-TQ-MS (quantification). Up to now, most studies have focused on ester-degrading activity by LAB using tributyrin (lipases [49, 52, 56]), and *p*-nitrophenyl butyrate or β-naphthyl butyrate for their assays (esterases [45–47, 50, 51, 57]). Only one study has employed ethanol and free fatty acids that were added to modified de Man-Rogosa-Sharpe (mMRS-5) medium, and ester biosynthesis activity by the wine strains tested was shown through HS/SPME-GC-TOF-MS (61). However, quantification of VOCs with LI-GC-TQ-MS is more accurate than analyses performed with HS/SPME-GC-TOF-MS. LI-GC-TQ-MS offers a higher accuracy as a result of the injection of the sample in liquid form (LI) compared to HS/SPME, which relies on the absorption of the VOCs on a fiber in the vial’s headspace, and for which parameters such as binding capacity, fiber type, and fiber saturation levels hamper a quantitative analysis. Moreover, LI-GC-TQ-MS offers a higher selectivity and accuracy, thanks to the triple-quadrupole mass spectrometer (TQ-MS) compared with a single mass spectrometer (TOF-MS). The fact that only ethyl acetate and ethyl lactate were produced in concentrations high enough to be quantified by LI-GC-TQ-MS during the mWSSM fermentation processes of the present study suggested that ester biosynthesis by the LAB strains tested during sourdough production will depend on the concentrations of the necessary precursor molecules. The latter can be synthesized *de novo*, but given the low ATP production and biomass yield of LAB during fermentation, their concentrations will be (too) low to achieve significant ester formation. Alternatively, the precursors can be released into the medium upon, for instance, yeast metabolite production and subsequent cell lysis, and have then to be taken up by the LAB cells. An increased ester production following the addition of the appropriate precursors has been shown for yeast strains before (62, 96, 97). Although ethyl acetate and ethyl lactate can also be produced chemically, as has been shown during lambic beer production (98), and as occurred during the uninoculated WSSM and mWSSM fermentation processes of the present study, their concentrations were significantly higher during fermentation processes carried out with specific LAB strains. The esterases EstA and/or EstC could be responsible for the biosynthesis of ethyl acetate and ethyl lactate, as the concomitant genes were not present in the genomes of the *Frul. sanfranciscensis* strains tested during the present study and for which no ester production could be shown. Moreover, at least one of these genes was present in the genomes of all LAB strains capable of ester biosynthesis. However, other yet unknown ester biosynthesis enzymes could likely be involved too, as well as other described esterases not yet studied as to their ester biosynthesis capacity in sourdoughs (99). Therefore, further research is needed to link the role of each enzyme with ester biosynthesis activity by LAB strains in general and sourdough LAB strains in particular. Although the *in vivo* ester biosynthesis by the esterases EstA, EstB, and EstC has been shown before (48), their role during sourdough production has not been studied yet. Experimental setups involving deletion of one or several of these genes in sourdough strains, or transcriptomic studies, followed by quantification of the fruity esters produced could give insights into the link between the genes and fruity ester productions. The production of sourdoughs in the presence of ester precursor molecules and started with one of these strains could reveal if the production of fruity esters also happens. Finally, in addition to the limited concentrations of the necessary ester precursors present in a sourdough matrix, different fermentation conditions could have an influence as well, such as the growth phase (60), the pH (48), or the water activity (48). Therefore, the process parameters should be carefully studied to be able to steer sourdough productions toward enhanced ester formation appropriately.

As fruity esters have a low sensory threshold, the ester concentrations synthesized by sourdough LAB as reported in the present study could presumably be detected if present in a sourdough-containing baked product. For example, ethyl acetate is detected at concentrations of over 7.5 mg/L in wine and 30.0 mg/L in beer (100). As fruity esters are often present in low concentrations in the bread crumb, ester detection thresholds are not available for sourdough-containing baked goods, although esters are positively valued in sensory analyses (8). Moreover, they should survive the baking process, during which evaporation losses will occur due to their high volatility (3). For example, it has been shown that short-chain acetate esters do not survive the chocolate making process (101). Yet, longer and more fat-soluble ethyl and acetate esters remain almost unaffected and, hence, contribute to the fruity flavor of chocolates.

Ester production by yeast species has been associated with the dispersion of strains, as the fruitiness of esters attracts insects (28). Therefore, their production influences the fitness of each strain in specific environments and could explain the strain dependency of the presence of ester biosynthesis genes in yeast species. The absence of the ester biosynthesis genes targeted and, hence, the lack of ester biosynthesis by *Frul. sanfranciscensis* strains could be ascribed to its small genome, which is a result of domestication and speciation to a sourdough environment (102). The reason why other specific LAB species synthesize esters is not known yet. The fact that esters are produced only when certain concentrations of the ester precursors occur, could indicate that the esters are produced either as part of a communication strategy or as a detoxification step when the concentrations of some organic acids or (higher) alcohols increase above a certain threshold in a specific environment. However, further research is needed to determine the minimal concentrations to trigger this biosynthesis.

Ester biosynthesis by LAB during sourdough production is difficult to unravel, given the presence of yeasts, although the presence of esters could be the result of both LAB and yeast involvement. Indeed, several studies have suggested the production of a higher number of esters or different ester profiles in sourdoughs produced with different starter cultures (11, 66–69, 71, 103–105), but in only two studies yeast growth was inhibited (66, 68). The limited availability of ester precursors during sourdough productions started with LAB strain(s) solely could be overcome by the use of mixed-strain starter cultures. Moreover, during sourdough production, yeasts and LAB interact, either positively or negatively, which influences the characteristics of the end-products too (106). For instance, the use of cocultures composed of LAB and yeast strains for bread dough fermentation results in different metabolite profiles and dough heights compared with those of the concomitant monocultures. Moreover, different VOC profiles were obtained during coculture fermentation processes compared with monoculture ones, as well as during monoculture fermentation processes involving one strain and cell-free extract of the other one (107). This way, the combination of the biosynthetic activities of LAB and yeast strains and the lysis of cells of the latter could result in the production of enough ester precursors for the LAB involved. Consequently, all together, this could lead to ester biosynthesis by both microorganisms and to different flavor profiles. Indeed, different ester profiles, characterizing sourdoughs and sourdough-containing baked products, occur when mixed-strain starter cultures are used instead of single-strain starter cultures (108, 109).

To conclude, the present study showed that both sourdough yeast and LAB strains possessed ester biosynthesis potential. Concerning the yeast strains, a screening strategy, based on a genome mining approach followed by PCR assays targeting either acetate ester or ethyl ester biosynthesis genes, was developed. This could be used for the selection of yeast strains for the production of fermented foods and beverages based on their ester biosynthesis potential. Concerning the LAB strains, a genome mining approach targeting three esterase genes showed the potential of ester biosynthesis by strains of specific LAB species. Moreover, a phenotypical assay showed the production of ethyl acetate and ethyl lactate by certain sourdough LAB strains under the appropriate fermentation conditions. Therefore, this phenotypical assay could be used to screen candidate starter culture LAB strains for fermented food and beverage production, in particular sourdoughs, as to their ester biosynthesis capacity. Nevertheless, the ester biosynthesis by LAB seemed to be limited by the concentrations of the ester precursors available in the fermentation medium. A way to overcome this limitation during sourdough production could be the use of both ester-producing yeast and LAB strains, whose metabolic activities and/or lysis could result in the formation of specific ester precursor and ester molecules that yield sourdoughs with an enhanced fruity flavor.

## MATERIALS AND METHODS

### Genome retrieval and phylogenetic tree constructions

First, a listing of yeast and LAB species names that are encountered in spontaneous backslopped sourdough productions was made based on literature data (110). Species with a genome available in the NCBI Genome database were selected. For strains that were isolated from sourdough, all genomes available were retrieved regardless of the genome assembly level. For strains not isolated from sourdough, complete genomes were preferred, and if not available, a genome at another assembly level was selected (Tables S1 and S2).

Subsequently, phylogenetic trees were constructed to better visualize the genome mining results (see “*In silico* genome mining and clustering analysis,” below). For the yeast genomes, this was performed based on amino acid sequences. Hereto, genes were predicted using AUGUSTUS relying on the *Saccharomycetes* database (111), and the amino acid sequences were inferred from the genes found. OrthoFinder (112) was used to search for orthogroups among these amino acid sequences and to build a phylogenetic tree. *Mucor circinelloides* JCM 22480 (NCBI accession number GCA_001599575.1) was used as an outgroup. The phylogenetic tree was visualized using *anvi-interactive* of anvi’o (113). For the LAB genomes, the phylogenetic analysis was performed at gene level, using the anvi’o phylogenomic workflow (113). Briefly, single-copy genes present in the bacterial genomes under study were selected using the Bacteria_71 (containing 71 single-copy genes) hidden Markov models profile of single-copy core genes for bacteria as reference database. Then, inferred distances from the approximately-maximum-likelihood of the alignments of their nucleotide sequences were used. *Leuconostoc mesenteroides* ATCC 8293 (accession number GCA_000014445.1) was used as an outgroup. The phylogenetic tree was visualized using *anvi-interactive* of anvi’o.

### *In silico* genome mining and clustering analysis

The presence of seven yeast genes (*ATF1*, *ATF2*, *EEB1*, *EHT1*, *YMR210w*, *EAT*, and *IMO32* [35, 40–44]), which have been linked to the production of fruity esters, such as isoamyl acetate, ethyl acetate, ethyl butanoate, ethyl hexanoate, ethyl octanoate, and/or ethyl decanoate, was searched for in the yeast genomes retrieved as mentioned above, using tblastn (114). Likewise, three LAB genes (*estA*, *estB*, and *estC* [45–47]), which have been linked to the production of ethyl butanoate, ethyl pentanoate, and/or ethyl hexanoate (48), were searched for in the LAB genomes using the same approach. Only tblastn hits with an E-value < 0.001, a query coverage >80%, and a sequence identity >30% were considered (115). For the LAB genomes, an additional, less stringent analysis was performed, in which only tblastn hits with an E-value < 0.001, a query coverage >80%, and a sequence similarity >30% were considered.

To target the abovementioned yeast genes in the yeast genomes, the amino acid sequence of the corresponding enzyme of the yeast-type strain (*S. cerevisiae* S288C) was used as query for the tblastn searches (accession numbers NP_015022.3, NP_011693.1, Q02891, NP_009736.3, NP_013937.1, DAA08112.1, and NP_011545.1 for the respective genes mentioned above). In addition, the amino acid sequence of the Eat acetyl transferase of *W. anomalus* RRL Y-366-8 was used (accession number XP_019041020.1).

To target the abovementioned LAB genes in the LAB genomes, the amino acid sequence of the three esterases of the LAB strain in which each of them was first described was used as query for the tblastn searches, namely, the EstA esterase of *L. helveticus* (accession number WP_003627824.1), and the EstB and EstC esterases of *Lacc. paracasei* (accession numbers CCK23986.1 and CAQ65481.1, respectively). Next, the functional annotation of these matching genomic regions was checked.

### PCR assays for the detection of ester biosynthesis genes in yeast genomic DNA

#### Optimization of the PCR assays

Two PCR assays were developed to detect the ester biosynthesis genes mentioned above in yeast genomic DNA. The first one targeted a 200–600 bp region of the acetate ester biosynthesis genes (*ATF1/ATF2*), and the second one targeted a region of the same size of the ethyl ester biosynthesis genes (*EEB1*/*EHT1*). To design specific primers, text searches in the NCBI Protein database, using the enzyme names as query, were used to collect, per enzyme, the amino acid sequences (Table S4). These sequences were back translated to nucleotide sequences using the EMBOSS Backtranseq tool (https://www.ebi.ac.uk/jdispatcher/st/emboss_backtranseq [116]), and a multiple sequence alignment (MSA) was subsequently performed using the multiple sequence comparison by log-expectation (MUSCLE) algorithm (https://www.ebi.ac.uk/jdispatcher/msa/muscle [117]). The consensus sequences of the MSAs were retrieved using EMBOSS cons (https://www.ebi.ac.uk/jdispatcher/msa/emboss_cons [116]), and they were used to design the primer sets using the Primer3 software (version 4.1.0 [118]), without allowing ambiguity. The primer set designed for the *ATF1/ATF2* PCR assays comprised the forward primer 5′-ATGTACTTTGACTCCATTTT-3′ and the reverse primer 5′-ACCAACATTAGACAACAAAGT-3′. The primer set designed for the *EEB1*/*EHT1* PCR assays comprised the forward primer 5′-GGTGGTGTTTGTACTGCTGA-3′ and the reverse primer 5′-CCAGCCAAACCATGCAAAAT-3′. The concentrations of the primers, the melting temperature applied, and the concentration of bovine serum albumin (BSA) to be used were optimized in a TProfessional Basic Thermocycler (Biometra, Göttingen, Germany), using the genomic DNA of *S. cerevisiae* MUCL 38902 that possessed the genes targeted (NCBI Nucleotide database). The amplification of the internal transcribed spacer (ITS) region (ITS1-ITS2) of the fungal rRNA transcribed unit, using the primers ITS1 and ITS4 (119), was used as a positive control during the optimization of these PCR assays. The optimized PCR conditions consisted of an initial denaturation of 2 min at 95°C, 30 cycles of 20 s at 95°C, 60 s at 41°C, and 30 s at 72°C, followed by a final elongation of 7 min at 70°C. The PCR amplicons obtained were visualized through gel electrophoresis, using 1.5% (m/vol) agarose gels and performed at 100 V for 1.5 h. The PCR amplicons were purified using the Wizard Plus SV Mini-preps DNA purification system (Promega, Madison, WI, USA). They were sequenced using Sanger technology (Macrogen, Amsterdam, The Netherlands).

#### PCR screening of sourdough yeast isolates

The optimized PCR assays were used to screen 91 sourdough yeast strains, belonging to 15 different species, which were available in the laboratory collection of the research group of Industrial Microbiology and Food Biotechnology (IMDO-VUB) and the Belgian Co-ordinated Collection of Microorganisms (BBCM/MUCL), as to the presence of the ester biosynthesis genes mentioned above (Table S3). These strains have been isolated from both bakery and laboratory sourdoughs produced in Belgium, France, the UK, or the USA. These strains were stored at −80°C in yeast extract-peptone-glucose (YPG) medium (5 g/L of yeast extract [Merck, Darmstadt, Germany], 10 g/L of bacteriological peptone [Oxoid, Basingstoke, Hampshire, UK], and 20 g/L of glucose [Merck]), supplemented with 25% (vol/vol) glycerol (Sigma-Aldrich, St. Louis, MO, USA) as cryoprotectant.

In view of the actual PCR screening, each yeast strain was grown on YPG agar medium at 30°C for 48 h. A single colony of the strains was transferred to 5 mL of liquid YPG medium and grown at the same temperature for 24 h. Two milliliters of these cultures was centrifuged at 10,000 × *g* for 10 min to obtain cell pellets, which were washed with Tris-ethylene diamine tetraacetic acid (EDTA)-sucrose (TES) buffer (50 mM Tris base [Calbiochem, Darmstadt, Germany], 1 mM EDTA [Sigma-Aldrich], and 6.7% [m/vol] sucrose [VWR International, Darmstadt, Germany]; pH 8.0). The cell pellets were used for genomic DNA extraction with a NucleoSpin 96 tissue kit (Macherey Nagel, Düren, Germany), as described previously (120). Subsequently, the genomic DNA of each strain was used as a template for PCR amplification following the optimized conditions mentioned above. The presence or absence of a PCR amplicon related to the gene region targeted was visualized by gel electrophoresis, using 1.5% (m/vol) agarose gels and performed at 100 V for 1.5 h.

### LAB genome sequencing and *in silico* genome mining for the detection of ester biosynthesis genes

A selection of 12 LAB strains from the IMDO-VUB laboratory collection, all isolated from sourdough before, was enabled for genome sequencing (Table 1). All strains were kept at −80°C in mMRS-5 medium (121), supplemented with 25% (vol/vol) glycerol as cryoprotectant.

#### Genomic DNA extraction

In view of genomic DNA extraction, each LAB strain was grown on mMRS-5 agar medium at 30°C for 48 h. A single colony of the strains was transferred to 10 mL of liquid mMRS-5 medium and grown at the same temperature for 24 h; 1% (vol/vol) of these cultures was used to inoculate 20 mL of the same medium, followed by incubation at 30°C for 24 h. The bacterial cell pellets were obtained by centrifugation of the latter cultures at 4,500 × *g* for 20 min.

DNA was extracted using the Qiagen Genomic-tip 20/G kit (Qiagen, Düsseldorf, Germany) following the manufacturer’s instructions with minor modifications. The bacterial cell pellets were resuspended in 1 mL of buffer B1 and supplemented with 2 µL of RNase A (10 mg/mL; Thermo Fisher Scientific, Waltham, MA, USA), 20 µL of a lysozyme solution (100 mg/mL; Merck), 8 µL of a mutanolysin solution (12.5 units/mL; Sigma-Aldrich), and 45 µL of a proteinase K solution (20 mg/mL, Merck), and were incubated at 37°C for 45 min. Subsequently, 350 µL of buffer B2 was added, and the mixture was incubated at 50°C for 45 min. The resulting lysate was centrifuged at 1,000 × *g* for 10 min and used for DNA purification, following the manufacturer’s instructions. The concentration of the DNA extracted was quantified using the dsDNA HS assay kit for Qubit (Thermo Fisher Scientific), and its quality was assessed using a Nanodrop 2000 spectrophotometer (Thermo Fisher Scientific).

#### Whole-genome sequencing and *de novo* genome assembly

Whole-genome sequencing of the LAB DNA was performed using a combination of long-read and short-read sequencing technologies. Long-read sequencing was performed by means of the ONT MinION sequencing device (Littlemore, Oxford, UK). To this end, 2–3 µg of extracted DNA of each LAB culture was used for library preparation using a NEBNext Companion Module for ONT Ligation Sequencing (New England Biolabs, Ipswitch, MA, USA), a SQK-LSK106 ONT ligation sequencing kit (ONT), and an EXP-NBD104 native barcoding expansion kit (ONT), according to the manufacturer’s instructions, with minor modifications. Briefly, the barcode and adapter ligation incubation times were extended to 20 and 30 min, respectively. Each sequencing run was performed with a pool of barcoded genomic DNA of 6 to 10 strains on either a flow cell R9.4 or R10.3 of the MinION MK1b device (ONT), using the MinKNOW software for data acquisition. Basecalling and barcode separation were performed using Guppy v4 in high-accuracy GPU-accelerated mode (ONT) with the dna_r9.4.1_450bps_hac.cfg (R9.4 flow cells) or the dna_r10.3_450bps_hac.cfg (R10.3 flow cells) configuration files. Quality check and trimming were carried out using NanoQC and NanoFilt, respectively, from Nanopack (122), using the following parameters: headcrop, 80; tailcrop, 60; quality >8, being specifically chosen for each strain based on the quality plot retrieved from NanoPlot. Paired-end short-read sequencing was performed using a NovaSeq platform (Illumina, San Diego, CA, USA) by the university’s core facility BRIGHTcore (Jette, Belgium). Quality check and trimming of the short reads were performed using FastQC (v0.11.3; http://www.bioinformatics.babraham.ac.uk/projects/fastqc/) and Trimmomatic (v0.36 [123]), respectively.

The short and long reads were used to assemble the genome of each LAB strain, developing a *de novo* hybrid assembly, using Unicycler v0.4.7, which was run in conservative mode (https://github.com/rrwick/Unicycler). The assessment of the quality of the final LAB genomes as well as their graphical representation was performed using Bandage (124).

#### *In silico* genome mining

Detection of the esterase-encoding genes *estA*, *estB*, and *estC* in the 14 selected IMDO LAB genomes (Table 1) was performed using tblastn. The same amino acid sequences described in “*In silico* genome mining and clustering analysis,” above, were used as query sequences.

### Phenotypic screening of LAB strains

Eight LAB strains, selected from the initial pool (Table 1), were further studied as to their ester biosynthesis capacity, namely, *Coml. crustorum* LMG 23699, *Coml. nantensis* R19088, *Coml. paralimentarius* 1Mg86, 1Mg105, BBRM18, and EBRM1, *Levl. zymae* LMG 22198, and *Liml. fermentum* IMDO 130101. The strains of *Frul. sanfranciscensis* ACA-DC 3378 and LMG 16002^T^ were included as negative controls based on the results obtained (see “Lactic acid bacteria,” above).

#### Microbial growth in wheat sourdough simulation medium (WSSM) and modified WSSM (mWSSM)

First, the growth potential of the LAB strains mentioned above was monitored in WSSM (125) and in mWSSM at 30°C for 28 h. WSSM contained 0.5 g/L of fructose (Merck), 0.5 g/L of glucose (Merck), 10.0 g/L of maltose (Merck), 2.0 g/L of sucrose (Merck), 12.0 g/L of wheat peptone (Merck), 12.0 g/L of yeast extract (Oxoid), 4.0 g/L of dipotassium phosphate (Merck), 0.2 g/L of magnesium sulfate (Merck), 0.05 g/L of manganese (II) sulfate (Merck), 4.0 g/L of potassium phosphate (Merck), 1.0 mL/L of Tween 80 (Sigma-Aldrich), and 1 mL/L of a vitamin solution (cobalamine, folic acid, nicotinamide, pantothenic acid, pyridoxal-phosphate, and thiamine; 0.2 g/L each). The mWSSM was obtained by the addition of an ester precursor mixture to WSSM. This ester precursor mixture consisted of ethanol (1.0%, vol/vol), acetic acid (0.5%, vol/vol), the organic acids propionic acid, butyric acid, pentanoic acid, hexanoic acid, decanoic acid, and lactic acid (5.0 mg/L each), and the higher alcohols 1-propanol, 1-butanol, 1-pentanol, 1-hexanol, and isoamyl alcohol (5.0 mg/L each). Therefore, a single colony of each strain was grown in 5 mL of liquid mMRS-5 medium at 30°C for 24 h. These liquid cultures were used to inoculate (1.0%, vol/vol) 100 mL of WSSM or mWSSM in Scott bottles (VWR International), in duplicate. To draw a growth curve, the optical density at 600 nm was measured (Genesys 20; Sigma-Aldrich) every 2 h. R package GrowthCurver (126) was used to fit the growth of each strain with the logistic equation


$$N_{t}=\frac{K}{1+\left(\frac{K-N_{0}}{N_{0}}\right)e^{-rt}}$$

where *N*_*t*_ is the population size at time *t*, *N*_0_ is the initial population size, *K* is the maximum population size in the particular environment, and *r* is the growth rate that would occur if there were no restrictions imposed on the total population size.

### Ester biosynthesis capacity

The ester biosynthesis capacity of each LAB strain was assessed during another series of fermentation processes carried out in WSSM and mWSSM under the conditions described above. Samples were taken every 3 h during the exponential growth phase and the beginning of the stationary phase. At every time point, the pH of the sample was measured with an InoLab 720 pH meter (WTW, Weilheim, Germany) immediately after withdrawing. Then, the samples were centrifuged at 5,000 × *g* for 10 min, and the supernatants were stored at −20°C until further analysis. These fermentation processes were carried out in duplicate.

A qualitative VOC fingerprinting analysis was performed, using 0.5 mL of each sample and applying HS/SPME-GC-TOF-MS. Therefore, a Trace 1300 gas chromatograph (Thermo Fisher Scientific) equipped with a fused silica capillary Stabilwax-MS column (Restek, Lisses, France) and coupled to a Bench TOF-HD mass spectrometer (Markes International, Llantrisant, Wales, UK) was used. Ten microliters of a 40 ppm solution of 4-methyl-2-propanol (Sigma-Aldrich) was added as internal standard (IS) to each sample. The VOC fingerprints of all samples were determined, in duplicate, as described previously (127).

A quantitative analysis of selected esters (allyl acetate, ethyl acetate, ethyl butanoate, ethyl decanoate, ethyl dodecanoate, ethyl hexanoate, ethyl lactate, ethyl pyruvate, ethyl octanoate, isoamyl acetate, hexyl acetate, isobutyl acetate, and pentyl acetate) was performed by means of LI-GC-TQ-MS, as described previously (128). Therefore, a Trace 1300 gas chromatograph equipped with a DBwax-UI column (Agilent, Santa Clara, CA, USA) coupled to a TSQ 8000 EVO triple-quadrupole mass spectrometer (Thermo Fisher Scientific) was employed. For sample preparation, 100 µL of the appropriate thawed supernatants was mixed with 900 µL of an IS solution (50 µg/L of deuterated diacetyl, deuterated ethyl decanoate, and deuterated 3-methyl-1-butanol in acetone; all from CDN Isotopes, Pointe-Claire, Quebec, Canada), mixed for 5 min, and microcentrifuged at 14,000 rpm for 15 min. Finally, the samples were filtered with 0.2 µm filters (Millex LG filter unit, Merck), collected into 0.4 mL glass vials (Macherey Nagel) with screw caps (Macherey Nagel), and injected (1.2 µL) into the column. All measurements were performed in duplicate.

### Statistical analysis

All statistical tests were performed with the RStudio software (version 3.5.2 [129]). In the case of duplicate experiments, averages were calculated. The data obtained from the HS/SPME-GC-TOF-MS were normalized according to the area of the peaks of the IS, and z-score transformations were calculated. Heatmaps created to visualize the presence/absence of genes of ester biosynthesis were generated using the ComplexHeatmap package (130).

## Data Availability

The whole genome sequences obtained during the current study are available at the European Nucleotide Archive of the European Bioinformatics Institute (ENA/EBI) under the BioProject accession number PRJEB64291. The accession numbers of the individual genomes are indicated in Table 1.

## SUPPLEMENTAL MATERIAL

Supplemental material aem.02216-24-s0001.pdf Tables S1 to S4; Figures S1 and S2.

ASM does not own the copyrights to Supplemental Material that may be linked to, or accessed through, an article. The authors have granted ASM a non-exclusive, world-wide license to publish the Supplemental Material files. Please contact the corresponding author directly for reuse.
