# Supplementary material for: The production of esters by specific sourdough lactic acid bacteria species is limited by the precursor concentrations
Source: Appl Environ Microbiol. 2025 Feb 27;91(3):e02216-24. doi: 10.1128/aem.02216-24 (PMC11921326; doi:10.1128/aem.02216-24)
Supplement: Supplemental material — Tables S1 to S4; Figures S1 and S2. [file aem.02216-24-s0001.pdf]

Supplementary tables

Table S1. Yeast genomes used for a genome mining analysis targeting genes for the biosynthesis of acetate esters (*ATF1/ATF2*), ethyl esters (*EHT1/EEB1/YMR210w*), and ethyl acetate (*EAT/IMO32*), and the number of genes obtained. NA, isolation source not available in the National Center for Biotechnology Information (NCBI) Genome database (https://www.ncbi.nlm.nih.gov/genome) at the time of analysis.

Strain	Genome Accession Number	Isolation source	*ATF1/ATF2*	*EHT1/EEB1/YMR210w*	*EAT/IMO32*
Candida intermedia CBS 141442	GCA_900106115.1	Others	0	2	0
Candida intermedia PYCC 4715	GCA_900106125.1	Environment	0	1	0
Candida tropicalis MYA 3404	GCA_013177555.1	Human	0	2	2
Debaryomyces hansenii CBS 767	GCA_000006445.2	NA	0	2	1
Debaryomyces hansenii Y-3	GCA_003349505.1	Sourdough	0	2	0
Dekkera anomala CRL 49	GCA_012295365.1	Beer	0	2	0
Dekkera anomala YV396	GCA_000340765.1	NA	0	0	0
Kazachstania aerobia NRRLY 27976	GCA_003708495.1	NA	1	1	1
Kazachstania barnettii CLIB 1767	GCA_903064755.1	Sourdough	1	2	2
Kazachstania barnettii CLIB 433	GCA_903987035.1	Environment	1	2	2
Kazachstania bulderi KABU0	GCA_933962305.1	Sourdough	1	2	2
Kazachstania exigua OG2	GCA_016584175.1	Fermented dairy product	1	2	2
Kazachstania humilis KAHU0	GCA_933934105.1	Sourdough	1	2	2
Kazachstania saulgeensis CLIB 1764^{T}	GCA_900180425.1	Sourdough	1	2	2
Kazachstania servazzii UCD 13	GCA_008802765.1	Environment	1	2	2
Kazachstania servazzii UCD 335	GCA_008802755.1	Environment	1	2	2
Kazachstania telluris UCD 400	GCA_009394695.1	Environment	1	2	2
Kazachstania unispora NRRLY 1556	GCA_003708525.2	NA	0	2	2
Kluyveromyces marxianus DMKU 3-1042	GCA_001417885.1	NA	1	2	2
Kluyveromyces marxianus FIM1	GCA_001854445.2	Fermented dairy product	1	2	2
Kluyveromyces marxianus KCTC 17555	GCA_000299195.2	Other foods	1	2	2
Kluyveromyces marxianus NRRLY 6860	GCA_002356615.1	Fruit	1	2	2
Kluyveromyces marxianus B0399	GCA_001660455.1	Fermented dairy product	1	2	2
Kluyveromyces marxianus NBRC 1777	GCA_001417835.1	NA	1	2	2
Kluyveromyces marxianus NRRLY 6860	GCA_002356615.1	Others	1	2	2
Metschnikowia pulcherrima Bath1	GCA_009932455.1	Fruit	0	1	2
Meyerozyma carpophila JCM 9396	GCA_001599235.1	NA	0	2	1
Meyerozyma guilliermondii ATCC 6260	GCA_000149425.1	NA	0	2	1
Meyerozyma guilliermondii MeyPP1	GCA_900174495.1	Animal	0	2	1
Meyerozyma guilliermondii SO	GCA_008062615.1	Other foods	0	2	1
Meyerozyma guilliermondii W2	GCA_008062615.1	Other foods	0	2	1
Meyerozyma guilliermondii YLG18	GCA_009756405.1	Environment	0	2	1

Nakaseomyces glabratus ATCC 2001	GCA_010111755.1	Human	1	2	1
Nakaseomyces glabratus CBS 138	GCA_000002545.2	NA	1	2	1
Nakaseomyces glabratus DSY 562	GCA_002219185.1	Human	1	2	1
Nakaseomyces glabratus DSY 565	GCA_002219195.1	Human	1	2	1
Naumovozyma castellii CBS 4309	GCA_000237345.1	NA	1	2	1
Pichia fermentans fo-MP-02	GCA_003339355.1	Fermented vegetable	0	3	2
Pichia kudriavzevii CBS 5147	GCA_003054405.1	Other foods	0	2	1
Pichia kudriavzevii CBS 573	GCA_003054445.1	Others	0	2	1
Pichia kudriavzevii SJP	GCA_003033855.1	NA	0	2	0
Pichia membranaefaciens S47-1	GCA_001950575.1	NA	0	2	1
Pichia norvegensis CBS 1922	GCA_003339405.1	Human	0	3	1
Pichia norvegensis NRRLY 7687	GCA_003705465.1	NA	0	2	1
Pichia occidentalis NRRLY 7552	GCA_003705455.2	NA	0	2	1
Rhodotorula mucilaginosa C2.5t1	GCA_000931965.1	Environment	0	0	0
Rhodotorula mucilaginosa IIPL32	GCA_002806785.1	Environment	0	0	0
Rhodotorula mucilaginosa JGTA-S1	GCA_003055205.1	Environment	0	0	0
Saccharomyces bayanus CBS380	GCA_013180695.1	NA	2	3	2
Saccharomyces bayanus FM1309	GCA_013180165.1	NA	2	3	2
Saccharomyces bayanus NCAIM676	GCA_013180065.1	Fermented drink	2	3	2
Saccharomyces bayanus NBRC 1948	GCA_013180125.1	NA	2	3	2
Saccharomyces cerevisiae S288C	GCA_000146045.2	NA	2	3	2
Saccharomyces cerevisiae BY4742	GCA_003086655.1	NA	2	3	2
Saccharomyces cerevisiae ySR128	GCA_004328465.1	NA	2	3	2
Saccharomyces cerevisiae ySR127	GCA_001051215.1	NA	2	3	2
Saccharomyces cerevisiae KSD-Yc	GCA_003709285.1	Fermented drink	2	3	2
Saccharomyces paradoxus UWOPS91-917.1	GCA_002079175.1	NA	2	3	2
Saccharomyces paradoxus UFRJ50816	GCA_002079145.1	NA	2	3	2
Saccharomyces paradoxus YPS138	GCA_002079115.1	NA	2	3	2
Saccharomyces paradoxus N44	GCA_002079085.1	NA	2	3	2
Saccharomyces paradoxus CBS 432	GCA_002079055.1	NA	2	3	2
Torulaspora delbrueckii COFT1	GCA_003013175.1	Wine	0	2	2
Torulaspora delbrueckii CBS 1146	GCA_000243375.1	NA	0	1	2
Torulaspora delbrueckii NRRLY 50541	GCA_001029055.1	Others	0	0	2
Wickerhamiella versatilis JCM 5958	GCA_001600375.1	NA	0	2	1
Wickerhamiella versatilis NRRLY 6652	GCA_004125065.1	NA	0	2	1
Wickerhamiella versatilis t-1	GCA_001659765.1	Others	0	1	1
Wickerhamomyces anomalus NRRLY 366	GCA_000147375.2	NA	2	0	6
Wickerhamomyces anomalus NRRLY 366-8	GCA_001661255.1	NA	1	1	3
Yarrowia lipolytica CLIB 89	GCA_003054345.1	Water	0	1	0

Yarrowia lipolytica CLIB 80	GCA_003054305.1	Environment	0	1	0
Yarrowia lipolytica DSM 3286	GCA_014490615.1	NA	0	1	1

Table S2. Lactic acid bacteria genomes used for a genome mining analysis targeting three esterase genes (*estA, estB, and estC*), and their presence (name of the gene) or absence (-) in each genome. NA, isolation source not available in the National Center for Biotechnology Information (NCBI) Genome database (https://www.ncbi.nlm.nih.gov/genome) at the time of analysis.

Strain	Genome Accession Number	Isolation source	Gene identity	Gene similarity
Amylolactobacillus amylophilus DSM 20533	GCA_001936335.1	Environment	*estA*	*estA*, *estC*
Companilactobacillus alimentarius DSM 20249	GCA_002849895.1	Fish	-	*estA*
Companilactobacillus crustorum JCM 15951	GCA_001434585.1	Sourdough	-	*estC*
Companilactobacillus crustorum LMG 23699	GCA_001438825.1	Sourdough	-	*estC*
Companilactobacillus crustorum MN 047	GCA_001951175.1	NA	-	*estC*
Companilactobacillus crustorum NBRC 107159	GCA_007992035.1	Sourdough	-	*estC*
Companilactobacillus farciminis DSM 20184	GCA_002706745.1	Meat	-	*estA*, *estC*
Companilactobacillus heilongijangensis DSM 28069	GCA_000831645.3	Fermented vegetable	-	*estA*, *estC*
Companilactobacillus kimchi KACC 12383	GCA_002191155.1	Kimchi	-	*estA*, *estB*, *estC*
Companilactobacillus mindensis DSM 14500	GCA_001434275.1	Sourdough	-	*estA*
Companilactobacillus mindensis NBRC 107162	GCF_007992055.1	Sourdough	-	*estA*
Companilactobacillus nantensis NBRC 107153	GCA_007991935.1	Sourdough	-	*estA*, *estC*
Companilactobacillus paralimentarius DSM 16982	GCA_001435815.1	Sourdough	-	*estA*, *estC*
Fructilactobacillus fructivorans LF 543	GCA_009496955.1	NA	-	-
Fructilactobacillus lindneri TMW 1.1993	GCF_001702115.1	Beer	-	-
Fructilactobacillus lindneri TMW 1.483	GCF_001702135.1	Beer	-	-
Fructilactobacillus sanfranciscensis Ah4	GCA_006334415.1	Sourdough	-	-
Fructilactobacillus sanfranciscensis DSM 20451	GCA_001436035.1	Sourdough	-	-
Fructilactobacillus sanfranciscensis Gs2	GCA_006334475.1	Sourdough	-	-
Fructilactobacillus sanfranciscensis Gs9	GCA_006334425.1	Sourdough	-	-
Fructilactobacillus sanfranciscensis JCM 5668	GCA_003369815.1	Sourdough	-	-
Fructilactobacillus sanfranciscensis Ls1001	GCA_009749295.1	Sourdough	-	-
Fructilactobacillus sanfranciscensis LS451	GCA_009496975.1	Sourdough	-	-
Fructilactobacillus sanfranciscensis Sd13	GCA_006334465.1	Sourdough	-	-
Fructilactobacillus sanfranciscensis TMW1.1150	GCA_002907325.1	Sourdough	-	-
Fructilactobacillus sanfranciscensis TMW1.1304	GCA_000225325.1	Sourdough	-	-
Fructilactobacillus sanfranciscensis TMW1.1597	GCA_002907285.1	Sourdough	-	-
Fructilactobacillus sanfranciscensis TMW1.2137	GCA_002907145.1	Sourdough	-	-
Fructilactobacillus sanfranciscensis TMW1.2138	GCA_002907155.1	Sourdough	-	-
Fructilactobacillus sanfranciscensis TMW1.2139	GCA_002907185.1	Sourdough	-	-
Fructilactobacillus sanfranciscensis TMW1.2140	GCA_002907205.1	Sourdough	-	-
Fructilactobacillus sanfranciscensis TMW1.2141	GCA_002907245.1	Sourdough	-	-
Fructilactobacillus sanfranciscensis TMW1.2142	GCA_002907225.1	Sourdough	-	-
Fructilactobacillus sanfranciscensis TMW1.392	GCA_002907345.1	Sourdough	-	-
Fructilactobacillus sanfranciscensis TMW1.53	GCA_002907365.1	Sourdough	-	-

Fructilactobacillus sanfranciscensis TMW1.54	GCA_002907305.1	Sourdough	-	-
Fructilactobacillus sanfranciscensis TMW1.726	GCA_002907255.1	Sourdough	-	-
Fructilactobacillus sanfranciscensis TMW11152	GCA_010377225.1	Sourdough	-	-
Fructilactobacillus sanfranciscensis TMW11154	GCA_010377205.1	Sourdough	-	-
Fructilactobacillus sanfranciscensis TMW11221	GCA_010377165.1	Sourdough	-	-
Fructilactobacillus sanfranciscensis TMW11304	GCA_010377175.1	Sourdough	-	-
Fructilactobacillus sanfranciscensis TMW11470	GCA_010377135.1	Sourdough	-	-
Fructilactobacillus sanfranciscensis TMW11730	GCA_010377125.1	Sourdough	-	-
Fructilactobacillus sanfranciscensis TMW12134	GCA_010377235.1	Sourdough	-	-
Fructilactobacillus sanfranciscensis TMW1640	GCA_010377275.1	Sourdough	-	-
Fructilactobacillus sanfranciscensis TMW1907	GCA_010377255.1	Sourdough	-	-
Fructilactobacillus sanfranciscensis Ts9	GCA_006334515.1	Sourdough	-	-
Furfurilactobacillus rossiae C5	GCA_009864005.1	Sourdough	-	-
Furfurilactobacillus rossiae DSM 15814	GCA_000428925.1	Sourdough	-	-
Furfurilactobacillus rossiae L1	GCA_013394005.1	Animal	-	-
Furfurilactobacillus rossiae L2	GCA_013394025.1	Fruit	-	-
Furfurilactobacillus rossiae L3	GCA_013394045.1	Fermented vegetable	-	-
Furfurilactobacillus rossiae L R814	GCA_009295885.1	Sourdough	-	-
Furfurilactobacillus rossiae RS	GCA_013395075.1	Sourdough	-	-
Lacticaseibacillus pantheris DSM 15945	GCA_001591865.1	NA	-	-
Lacticasebacillus saniviri DSM 24301	GCA_001437465.1	Human	-	*estA*, *estB*, *estC*
Lacticaseibacillus paracasei 10266	GCA_008329845.1	Human	*estB*, *estC*	*estA*, *estB*, *estC*
Lacticaseibacillus paracasei 34716	GCA_012955485.1	Human	*estB*, *estC*	*estA*, *estB*, *estC*
Lacticaseibacillus paracasei AO356	GCA_003957435.1	Human	*estB*, *estC*	*estA*, *estB*, *estC*
Lacticaseibacillus paracasei ATCC 334	GCA_000014525.1	NA	*estB*	*estA*, *estB*, *estC*
Lacticaseibacillus paracasei BDII	GCA_000194765.1	Fermented dairy product	*estB*, *estC*	*estA*, *estB*, *estC*
Lacticaseibacillus paracasei BL23	GCA_000026485.1	NA	*estB*, *estC*	*estA*, *estB*, *estC*
Lacticaseibacillus paracasei CACC566	GCA_009931715.1	Animal	*estB*, *estC*	*estA*, *estB*, *estC*
Lacticaseibacillus paracasei CAUH35	GCA_001191565.1	Fermented dairy product	*estB*, *estC*	*estA*, *estB*, *estC*
Lacticaseibacillus paracasei CBA3611	GCA_007292115.1	Human	*estB*, *estC*	*estA*, *estB*, *estC*
Lacticaseibacillus paracasei EG9	GCA_003177075.1	Fermented dairy product	*estB*, *estC*	*estA*, *estB*, *estC*
Lacticaseibacillus paracasei FAM18149	GCA_002442835.1	Fermented dairy product	*estB*, *estC*	*estA*, *estB*, *estC*
Lacticaseibacillus paracasei HD1.7	GCA_002865565.1	Fermented vegetable	*estB*, *estC*	*estA*, *estB*, *estC*
Lacticaseibacillus paracasei HDS01	GCA_002902825.1	Fermented vegetable	*estB*, *estC*	*estA*, *estB*, *estC*
Lacticaseibacillus paracasei IBB3423	GCA_009739485.1	NA	*estB*, *estC*	*estA*, *estB*, *estC*
Lacticaseibacillus paracasei IIA	GCA_002079285.1	Animal	*estB*, *estC*	*estA*, *estB*, *estC*
Lacticaseibacillus paracasei IJHSONE68	GCA_003966835.1	Fruit	*estB*, *estC*	*estA*, *estB*, *estC*
Lacticaseibacillus paracasei JCM 8130	GCA_000829035.1	NA	*estB*, *estC*	*estA*, *estB*, *estC*
Lacticaseibacillus paracasei KL1	GCA_001514415.1	Fermented dairy product	*estB*, *estC*	*estA*, *estB*, *estC*
Lacticaseibacillus paracasei L9	GCA_001244395.1	Human	*estB*, *estC*	*estA*, *estB*, *estC*

Lacticaseibacillus paracasei LC2W	GCA_000194785.1	Fermented dairy product	*estB*, *estC*	*estA*, *estB*, *estC*
Lacticaseibacillus paracasei LC355	GCA_003268715.1	Fermented dairy product	*estB*, *estC*	*estA*, *estB*, *estC*
Lacticaseibacillus paracasei LOCK919	GCA_000418515.1	NA	*estB*, *estC*	*estA*, *estB*, *estC*
Lacticaseibacillus paracasei Lp02	GCA_013307125.1	Human	*estB*, *estC*	*estA*, *estB*, *estC*
Lacticaseibacillus paracasei Lpc10	GCA_003199005.1	Wine	*estB*, *estC*	*estA*, *estB*, *estC*
Lacticaseibacillus paracasei MGYGHGUT02388	GCA_902386635.1	Human	*estB*, *estC*	*estA*, *estB*, *estC*
Lacticaseibacillus paracasei MN1115	GCA_000582665.1	Fermented dairy product	*estB*, *estC*	*estA*, *estB*, *estC*
Lacticaseibacillus paracasei NJ	GCA_007637635.1	Fermented dairy product	*estB*, *estC*	*estA*, *estB*, *estC*
Lacticaseibacillus paracasei SRCM 103299	GCA_004141835.1	Other foods	*estB*, *estC*	*estA*, *estB*, *estC*
Lacticaseibacillus paracasei TCS	GCA_008807095.1	Fermented dairy product	*estB*, *estC*	*estA*, *estB*, *estC*
Lacticaseibacillus paracasei TD062	GCA_009834405.1	Fermented dairy product	*estB*, *estC*	*estA*, *estB*, *estC*
Lacticaseibacillus paracasei TK1501	GCA_002257625.1	Other foods	*estB*, *estC*	*estA*, *estB*, *estC*
Lacticaseibacillus paracasei TMW1.1434	GCA_002813615.1	Fermented dairy product	*estB*, *estC*	*estA*, *estB*, *estC*
Lacticaseibacillus paracasei W56	GCA_000318035.1	NA	*estB*, *estC*	*estA*, *estB*, *estC*
Lacticaseibacillus paracasei ZFM54	GCA_003627255.1	Human	*estB*, *estC*	*estA*, *estB*, *estC*
Lacticaseibacillus paracasei Zhang	GCA_000019245.3	Fermented dairy product	*estB*, *estC*	*estA*, *estB*, *estC*
Lacticaseibacillus rhamnosus 1.032	GCA_006151905.1	Fermented dairy product	*estB*	*estA*, *estB*
Lacticaseibacillus rhamnosus 4B15	GCA_002158925.1	Human	*estB*	*estA*, *estB*
Lacticaseibacillus rhamnosus ATCC 11443 / JCM 1553	GCA_003433395.1	NA	*estB*	*estA*, *estB*
Lacticaseibacillus rhamnosus ATCC 8530	GCA_000233755.1	NA	*estB*	*estA*, *estB*
Lacticaseibacillus rhamnosus BFE5264	GCA_001988935.1	Fermented dairy product	*estB*	*estA*, *estB*
Lacticaseibacillus rhamnosus BIO5326	GCA_009720565.1	Human	*estB*	*estA*, *estB*
Lacticaseibacillus rhamnosus BIO6870	GCA_008831425.1	Human	*estB*	*estA*, *estB*
Lacticaseibacillus rhamnosus CECT 8800 / BPL5	GCA_900070175.1	Human	*estB*	*estA*, *estB*
Lacticaseibacillus rhamnosus DSM14870	GCA_002287945.1	NA	*estB*	*estA*, *estB*
Lacticaseibacillus rhamnosus GG / ATTC 53103	GCA_003353455.1	Human	*estB*	*estA*, *estB*
Lacticaseibacillus rhamnosus hsryfm1301	GCA_008727835.1	Human	*estB*	*estA*, *estB*
Lacticaseibacillus rhamnosus Lc705	GCA_000026525.1	NA	*estB*	*estA*, *estB*
Lacticaseibacillus rhamnosus LOCK900	GCA_000418475.1	NA	*estB*	*estA*, *estB*
Lacticaseibacillus rhamnosus LOCK908	GCA_000418495.1	NA	*estB*	*estA*, *estB*
Lacticaseibacillus rhamnosus LR5	GCA_002286235.1	Human	*estB*	*estA*, *estB*
Lacticaseibacillus rhamnosus LRB	GCA_001721925.1	Human	*estB*	*estA*, *estB*
Lacticaseibacillus rhamnosus LRB1	GCA_004010975.1	Human	*estB*	*estA*, *estB*
Lacticaseibacillus rhamnosus LV108	GCA_013167115.1	Human	*estB*	*estA*, *estB*
Lacticaseibacillus rhamnosus MGYGHGUT01293	GCA_902381635.1	Human	*estB*	*estA*, *estB*
Lacticaseibacillus rhamnosus NCTC 13710	GCA_900636875.1	NA	*estB*	*estA*, *estB*
Lacticaseibacillus rhamnosus NCTC 13764	GCA_900636965.1	NA	*estB*	*estA*, *estB*
Lacticaseibacillus rhamnosus Pen	GCA_002076955.1	Human	*estB*	*estA*, *estB*
Lacticaseibacillus rhamnosus SCT101060	GCA_002960215.1	Human	*estB*	*estA*, *estB*
Lacticaseibacillus zeae CECT 9104	GCA_900492555.1	Human	*estB*	*estB*

Lactiplantibacillus pentosus BGM48	GCA_002850015.1	Fermented vegetable	-	*estC*
Lactiplantibacillus pentosus DSM 20314	GCA_003641185.1	NA	-	*estC*
Lactiplantibacillus pentosus SLC13	GCA_002211885.1	Fermented vegetable	-	*estC*
Lactiplantibacillus pentosus ZFM222	GCA_003627295.1	Fermented vegetable	-	*estC*
Lactiplantibacillus pentosus ZFM94	GCA_003627375.1	Human	-	*estC*
Lactiplantibacillus plantarum 80	GCA_001368775.1	Cocoa	-	-
Lactiplantibacillus plantarum 8318	GCA_009759825.1	Human	-	-
Lactiplantibacillus plantarum 12317	GCA_009759845.1	Human	-	-
Lactiplantibacillus plantarum 202195	GCA_010586945.1	Human	-	-
Lactiplantibacillus plantarum 12_3	GCA_004028335.1	Fermented dairy product	-	-
Lactiplantibacillus plantarum 13_3	NZ_CP035020	Fermented dairy product	-	-
Lactiplantibacillus plantarum 8PA3	GCA_009762745.1	Human	-	-
Lactiplantibacillus plantarum AMT74419	GCA_012974545.1	Kimchi	-	-
Lactiplantibacillus plantarum BCC9546	GCA_014084065.1	Meat	-	-
Lactiplantibacillus plantarum BK021	GCA_013487805.1	Fermented vegetable	-	-
Lactiplantibacillus plantarum BNH17	GCA_005576935.1	Other foods	-	-
Lactiplantibacillus plantarum CACC558	GCA_010092485.1	Animal	-	-
Lactiplantibacillus plantarum CNEIKCA4	GCA_013155145.1	Other foods	-	-
Lactiplantibacillus plantarum CNEIKCA5	GCA_013694305.1	Other foods	-	-
Lactiplantibacillus plantarum DSM 16365	GCA_003641165.1	Other foods	-	-
Lactiplantibacillus plantarum DSM 20174	GCA_014131735.1	Fermented vegetable	-	-
Lactiplantibacillus plantarum EM	GCA_004337615.1	Kimchi	-	-
Lactiplantibacillus plantarum FBL3a	GCA_003999275.1	Animal	-	-
Lactiplantibacillus plantarum G1	GCA_013256965.1	Other foods	-	-
Lactiplantibacillus plantarum HC2	GCA_013458335.1	Fish	-	-
Lactiplantibacillus plantarum Heal19	GCA_013367715.1	Human	-	-
Lactiplantibacillus plantarum IRG1	GCA_004319665.1	Human	-	-
Lactiplantibacillus plantarum KACC92189	GCA_003692595.1	NA	-	-
Lactiplantibacillus plantarum KCCP11226	GCA_009720585.1	Kimchi	-	-
Lactiplantibacillus plantarum LLY606	GCA_006770505.1	Human	-	-
Lactiplantibacillus plantarum LMT148	GCA_003813125.1	Kimchi	-	-
Lactiplantibacillus plantarum LS-07	GCA_011304595.1	Human	-	-
Lactiplantibacillus plantarum NCIMB700965.EF.A	GCA_004328745.1	NA	-	-
Lactiplantibacillus plantarum nF1FD	GCA_003952885.1	Kimchi	-	-
Lactiplantibacillus plantarum pc26	GCA_006770485.1	Human	-	-
Lactiplantibacillus plantarum Q7	GCA_003999605.1	Fermented dairy product	-	-
Lactiplantibacillus plantarum SK156	GCA_014041895.1	Other foods	-	-
Lactiplantibacillus plantarum SPCSNU722	GCA_012109355.1	Kimchi	-	-
Lactiplantibacillus plantarum SRCM 100438	GCA_009913615.1	Human	-	-
Lactiplantibacillus plantarum SRCM 100440	GCA_009913635.1	Human	-	-

Lactiplantibacillus plantarum SRCM 100442	GCA_009913655.1	Human	-	-
Lactiplantibacillus plantarum SRCM 100995	GCA_009913675.1	Fermented vegetable	-	-
Lactiplantibacillus plantarum SRCM 101105	GCA_009913695.1	Kimchi	-	-
Lactiplantibacillus plantarum SRCM 101167	GCA_009914095.1	Kimchi	-	-
Lactiplantibacillus plantarum SRCM 101187	GCA_009913795.1	Kimchi	-	-
Lactiplantibacillus plantarum SRCM 101222	GCA_009913835.1	Kimchi	-	-
Lactiplantibacillus plantarum SRCM 101511	GCA_009937825.1	Kimchi	-	-
Lactiplantibacillus plantarum SRCM 101518	GCA_009913855.1	Kimchi	-	-
Lactiplantibacillus plantarum SRCM 102737	GCA_009913975.1	Other foods	-	-
Lactiplantibacillus plantarum SRCM 103295	GCA_004087995.1	Other foods	-	-
Lactiplantibacillus plantarum SRCM 103297	GCA_004141755.1	Other foods	-	-
Lactiplantibacillus plantarum SRCM 103300	GCA_004141875.1	Other foods	-	-
Lactiplantibacillus plantarum SRCM 103311	GCA_004101325.1	Other foods	-	-
Lactiplantibacillus plantarum SRCM 103357	GCA_004101505.1	Other foods	-	-
Lactiplantibacillus plantarum SRCM 103361	GCA_004101545.1	Other foods	-	-
Lactiplantibacillus plantarum SRCM 103362	GCA_004101605.1	Other foods	-	-
Lactiplantibacillus plantarum SRCM 103418	GCA_004101625.1	Other foods	-	-
Lactiplantibacillus plantarum SRCM 103472	GCA_004103495.1	Other foods	-	-
Lactiplantibacillus plantarum SRCM103473	GCA_004103515.1	Other foods	-	-
Lactiplantibacillus plantarum TCI507	GCA_013305265.1	Fruit	-	-
Lactiplantibacillus plantarum TMW1.1308	GCA_009619495.1	NA	-	-
Lactiplantibacillus plantarum UNQLp11	GCA_004730965.1	Wine	-	-
Lactiplantibacillus plantarum WLPL04	GCA_001331925.2	Human	-	-
Lactiplantibacillus plantarum X7022	GCA_011022295.1	Other foods	-	-
Lactiplantibacillus plantarum Y44	GCA_007833595.1	Fish	-	-
Lactiplantibacillus plantarum YW11	GCA_004028295.1	Fermented dairy product	-	-
Lactiplantibacillus plantarum ZFM4	GCA_003627355.1	Human	-	-
Lactiplantibacillus plantarum ZFM9	GCA_003627335.1	Human	-	-
Lactiplantibacillus xiangfangensis LMG 26013	GCA_001438845.1	NA	-	-
Lactobacillus acetotolerans CN247	GCA_009738535.1	Beer	*estA*	*estA*, *estC*
Lactobacillus acetotolerans LA749	GCA_008831485.1	Fermented drink	*estA*	*estA*, *estC*
Lactobacillus acetotolerans LJ49	GCA_012848655.1	Fermented drink	*estA*	*estA*, *estC*
Lactobacillus acetotolerans NBRC 13120	GCA_001042405.1	Fermented drink	*estA*	*estA*, *estC*
Lactobacillus acidophilus DSM 20079	GCA_003047065.1	Human	*estA*	*estA*, *estC*
Lactobacillus acidophilus LAG80111	GCA_013342945.1	NA	*estA*	*estA*, *estC*
Lactobacillus amylolyticus L5	GCA_003999355.1	Other foods	*estA*	*estA*
Lactobacillus amylolyticus L6	GCA_002075105.1	Other foods	*estA*	*estA*
Lactobacillus amylovorus 30SC	GCA_000191545.1	NA	*estA*	*estA*, *estC*
Lactobacillus amylovorus DSM 20531	GCA_002706375.1	Other foods	*estA*	*estA*, *estC*
Lactobacillus crispatus 1D	GCA_013487905.1	Animal	*estA*	*estA*, *estC*

Lactobacillus crispatus AB70	GCA_003971565.1	Human	*estA*	*estA*, *estC*
Lactobacillus crispatus B4	GCA_013456995.1	Human	*estA*	*estA*, *estC*
Lactobacillus crispatus CD21.1	GCA_009769205.1	Animal	*estA*	*estA*, *estC*
Lactobacillus crispatus CO3MRSI1	GCA_003795065.1	Human	*estA*	*estA*, *estC*
Lactobacillus crispatus FDAARGOS_743	GCA_009730275.1	Human	*estA*	*estA*, *estC*
Lactobacillus delbrueckii KCTC 13731	GCA_001888945.1	Environment	*estA*	*estA*, *estC*
Lactobacillus delbrueckii KLDS 1.0207	GCA_003597655.1	Fermented dairy product	*estA*	*estA*, *estC*
Lactobacillus delbrueckii NBRC 3202	GCA_006740305.1	Other foods	*estA*	*estA*, *estC*
Lactobacillus delbrueckii subsp. *bulgaricus* 2038	GCA_000191165.1	NA	*estA*	*estA*, *estC*
Lactobacillus delbrueckii subsp. *bulgaricus* ACA-DC 87	GCA_900196735.1	Fermented dairy product	*estA*	*estA*, *estC*
Lactobacillus delbrueckii subsp. *bulgaricus* ATCC 11842	GCA_000056065.1	NA	*estA*	*estA*, *estC*
Lactobacillus delbrueckii subsp. *bulgaricus* ATCC BAA365	GCA_000014405.1	NA	*estA*	*estA*, *estC*
Lactobacillus delbrueckii subsp. *bulgaricus* DSM20080	GCA_001953135.1	Environment	*estA*	*estA*, *estC*
Lactobacillus delbrueckii subsp. *bulgaricus* KLDS1.1011	GCA_006704185.1	Other foods	*estA*	*estA*, *estC*
Lactobacillus delbrueckii subsp. *bulgaricus* L99	GCA_003351805.1	Fermented dairy product	*estA*	*estA*, *estC*
Lactobacillus delbrueckii subsp. *bulgaricus* LJJ	GCA_011044195.1	Fermented dairy product	*estA*	*estA*, *estC*
Lactobacillus delbrueckii subsp.bulgaricus-ND02	GCA_000182835.1	NA	*estA*	*estA*, *estC*
Lactobacillus delbrueckii subsp. *bulgaricus* NMNBMF01	GCA_001469775.1	Fermented dairy product	*estA*	*estA*, *estC*
Lactobacillus delbrueckii subsp. *bulgaricus* ND04	GCA_002000885.1	Fermented dairy product	*estA*	*estA*, *estC*
Lactobacillus delbrueckii subsp. *jakobsenii* DSM 26046	GCA_001888925.1	Environment	*estA*	*estA*, *estC*
Lactobacillus delbrueckii subsp. *lactis* 1	GCA_900322585.1	Fermented dairy product	*estA*	*estA*, *estC*
Lactobacillus delbrueckii subsp. *lactis* DSM 20072	GCA_002278095.1	Fermented dairy product	*estA*	*estA*, *estC*
Lactobacillus delbrueckii subsp. *lactis* KCCM 34717	GCA_001888905.1	Environment	*estA*	*estA*, *estC*
Lactobacillus delbrueckii subsp. *lactis* KCTC 3034	GCA_002285775.1	Fermented dairy product	*estA*	*estA*, *estC*
Lactobacillus delbrueckii subsp. *lactis* KCTC 3035	GCA_001888985.1	NA	*estA*	*estA*, *estC*
Lactobacillus delbrueckii subsp. lactis NWC_1_2	GCA_003814285.1	Fermented dairy product	*estA*	*estA*, *estC*
Lactobacillus delbrueckii subsp. *sunkii* JCM 17838	GCA_001888965.1	Environment	*estA*	*estA*, *estC*
Lactobacillus delbrueckii TS106	GCA_009734125.1	Fermented dairy product	*estA*	*estA*, *estC*
Lactobacillus delbrueckii TUA4408L	GCA_002142575.1	Fruit	*estA*	*estA*, *estC*
Lactobacillus gallinarum HFD4	GCA_001314245.2	Human	*estA*	*estA*, *estC*
Lactobacillus helveticus CAUH18	GCA_001308285.1	Fermented dairy product	*estA*	*estA*, *estC*
Lactobacillus helveticus CNRZ 32	GCA_000422165.1	Fermented dairy product	*estA*	*estA*, *estC*
Lactobacillus helveticus D75	GCA_001746265.2	Human	*estA*	*estA*, *estC*
Lactobacillus helveticus D76	GCA_001702095.1	Human	*estA*	*estA*, *estC*
Lactobacillus helveticus FAM 22155	GCA_002849955.1	Other foods	*estA*	*estA*, *estC*
Lactobacillus helveticus FAM 8105	GCA_002849935.1	Fermented dairy product	*estA*	*estA*, *estC*
Lactobacillus helveticus FAM 8627	GCA_002849915.1	Fermented dairy product	*estA*	*estA*, *estC*
Lactobacillus helveticus H9	GCA_000525715.1	NA	*estA*	*estA*, *estC*
Lactobacillus helveticus IDCC 3801	GCA_004114755.1	Human	*estA*	*estA*, *estC*
Lactobacillus helveticus KLDS 1.8701	GCA_000961015.1	Fermented dairy product	*estA*	*estA*, *estC*

Lactobacillus helveticus LH99	GCA_003610975.1	Fermented dairy product	*estA*	*estA*, *estC*
Lactobacillus helveticus LZR5	GCA_009498395.1	Fermented dairy product	*estA*	*estA*, *estC*
Lactobacillus helveticus MB21	GCA_001006025.1	Fermented dairy product	*estA*	*estA*, *estC*
Lactobacillus helveticus MGYGHGUT02384	GCA_902386585.1	Human	*estA*	*estA*, *estC*
Lactobacillus helveticus NWC_2_3	GCA_003814305.1	Fermented dairy product	*estA*	*estA*, *estC*
Lactobacillus helveticus NWC_2_4	GCA_003814325.1	Fermented dairy product	*estA*	*estA*, *estC*
Lactobacillus helveticus R0052	GCA_000165775.3	Animal	*estA*	*estA*, *estC*
Lactobacillus johnsonii 3DG	GCA_013487865.1	Animal	*estA*	*estA*, *estB*, *estC*
Lactobacillus johnsonii BS15	GCA_001714745.1	Fermented dairy product	*estA*	*estA*, *estB*, *estC*
Lactobacillus johnsonii Byunjo01	GCA_003316915.1	Animal	*estA*	*estA*, *estB*, *estC*
Lactobacillus johnsonii DC22.2	GCA_009769185.1	Animal	*estA*	*estA*, *estB*, *estC*
Lactobacillus johnsonii G2A	GCA_010586925.1	Animal	*estA*	*estA*, *estB*, *estC*
Lactobacillus johnsonii IDCC 9203	GCA_003428395.1	Human	*estA*	*estA*, *estB*, *estC*
Lactobacillus johnsonii NCC 533	GCA_000008065.1	Human	*estA*	*estA*, *estB*, *estC*
Lactobacillus johnsonii NCK 2677	GCA_014058685.1	Animal	*estA*	*estA*, *estB*, *estC*
Lactobacillus johnsonii pf01	GCA_000219475.3	Animal	*estA*	*estA*, *estB*, *estC*
Lactobacillus johnsonii UMNLJ 21	GCA_002176855.1	Animal	*estA*	*estA*, *estB*, *estC*
Lactobacillus johnsonii UMNLJ 22	GCA_002176835.1	Animal	*estA*	*estA*, *estB*, *estC*
Lactobacillus johnsonii ZLJ010	GCA_004011315.1	Animal	*estA*	*estA*, *estB*, *estC*
Latilactobacillus curvatus CBA3617	GCA_007954645.1	Kimchi	-	-
Latilactobacillus curvatus DSM 20019	GCA_004101845.1	Fermented dairy product	-	-
Latilactobacillus curvatus FBA2	GCA_001663835.1	Other foods	-	-
Latilactobacillus curvatus IRG2	GCA_003957415.1	Human	-	-
Latilactobacillus curvatus KG6	GCA_002224505.1	Meat	-	-
Latilactobacillus curvatus MRS6	GCA_002224425.1	Meat	-	-
Latilactobacillus curvatus NFH-Km12	GCA_003966815.1	Environment	-	-
Latilactobacillus curvatus SPC-SNU 70-3	GCA_018732225.1	Sourdough	-	-
Latilactobacillus curvatus SRCM 103465	GCA_004088235.1	Other foods	-	-
Latilactobacillus curvatus TMW1.1928	GCA_003410375.1	Meat	-	-
Latilactobacillus curvatus Wikim38	GCA_001723545.1	Kimchi	-	-
Latilactobacillus curvatus WiKim52	GCA_001698165.1	Other foods	-	-
Latilactobacillus curvatus ZJUNIT8	GCA_003254785.1	Other foods	-	-
Latilactobacillus sakei LG 542	GCA_009025875.1	Environment	-	-
Latilactobacillus sakei CBA 3635	GCA_014081765.1	NA	-	-
Latilactobacillus sakei FLEC01	GCA_900234345.1	Human	-	-
Latilactobacillus sakei 23K	GCA_000026065.1	Meat	-	-
Latilactobacillus sakei CBA 3614	GCA_009676365.1	Kimchi	-	-
Latilactobacillus sakei CBA 3635	GCA_014081765.1	Kimchi	-	-
Latilactobacillus sakei DS4	GCA_002953655.1	Kimchi	-	-
Latilactobacillus sakei FAM 18311	GCA_002224565.1	Food	-	-

Latilactobacillus sakei J54	GCA_900234395.1	Meat	-	-
Latilactobacillus sakei J64	GCA_900234355.1	Meat	-	-
Latilactobacillus sakei LK145	GCA_002370375.1	Other foods	-	-
Latilactobacillus sakei LT13	GCA_002370355.1	Fermented drink	-	-
Latilactobacillus sakei LZ217	GCA_003627875.1	Fermented vegetable	-	-
Latilactobacillus sakei MBEL 1397	GCA_010092945.1	Kimchi	-	-
Latilactobacillus sakei MFPB 19	GCA_900234405.1	Food	-	-
Latilactobacillus sakei WiKim0072	GCA_003288195.1	Kimchi	-	-
Latilactobacillus sakei WiKim0074	GCA_003288235.1	Kimchi	-	-
Latilactobacillus sakei ZFM 220	GCA_003627275.1	Food	-	-
Latilactobacillus sakei ZFM 225	GCA_003627235.1	Food	-	-
Latilactobacillus sakei ZFM 229	GCA_003627315.1	Fermented vegetable	-	-
Latilactobacillus ultanensis Kx293C1	GCA_016647595.1	Human	-	*estA*, *estC*
Lentilactobacillus buchneri ATCC 4005	GCA_000211375.1	Other foods	-	-
Lentilactobacillus buchneri MGB0786	GCA_008369805.1	Kimchi	-	-
Lentilactobacillus diolivorans DSM 14421	GCA_001434255.1	Environment	-	-
Lentilactobacillus farraginis DSM 18382	GCA_001435875.1	Environment	-	*estC*
Lentilactobacillus hilgardii FLUB	GCA_009832765.1	Fermented drink	-	*estC*
Lentilactobacillus hilgardii LH500	GCA_008694025.1	Wine	-	*estC*
Lentilactobacillus hilgardii LMG 07934	GCA_011765585.1	Wine	-	*estC*
Lentilactobacillus kefiri DH5	GCA_009739535.1	Kefir	-	-
Lentilactobacillus otakiensis DSM 19908	GCA_001311395.1	NA	-	-
Lentilactobacillus parabuchneri FAM 21731	GCA_001922025.1	Fermented dairy product	-	*estC*
Levilactobacillus acidifarinae DSM 19394	GCA_001434835.1	Sourdough	-	-
Levilactobacillus acidifarinae NBRC 107156	GCA_007991975.1	Sourdough	-	-
Levilactobacillus brevis 100D8	GCA_002138395.1	Environment	-	*estC*
Levilactobacillus brevis ATCC 367	GCA_000014465.1	NA	-	*estC*
Levilactobacillus brevis BDGP6	GCA_002762175.1	Animal	-	*estC*
Levilactobacillus brevis KB290	GCA_000359625.1	NA	-	*estC*
Levilactobacillus brevis LMT173	GCA_003813165.1	Kimchi	-	*estC*
Levilactobacillus brevis NCTC 13768	GCA_900475625.1	NA	-	*estC*
Levilactobacillus brevis NPSQW145	GCA_001676805.1	Kimchi	-	*estC*
Levilactobacillus brevis SAC12	GCA_006228225.1	Environment	-	*estC*
Levilactobacillus brevis SRCM 101106	GCA_002174235.1	Other foods	-	*estC*
Levilactobacillus brevis SRCM 101174	GCA_002173555.1	Other foods	-	*estC*
Levilactobacillus brevis TMW1.2108	GCA_002117345.1	Beer	-	*estC*
Levilactobacillus brevis TMW1.2111	GCA_002117375.1	Beer	-	*estC*
Levilactobacillus brevis TMW1.2112	GCA_002117225.1	Beer	-	*estC*
Levilactobacillus brevis TMW1.2113	GCA_002117325.1	Beer	-	*estC*
Levilactobacillus brevis UCCLB 521	GCA_006228305.1	Beer	-	*estC*

Levilactobacillus brevis UCCLB 556	GCA_006228245.1	Beer	-	*estC*
Levilactobacillus brevis UCCLB 95	GCA_006228265.1	Beer	-	*estC*
Levilactobacillus brevis UCCLBBS 124	GCA_006228205.1	Beer	-	*estC*
Levilactobacillus brevis UCCLBBS 449	GCA_006228285.1	Beer	-	*estC*
Levilactobacillus brevis ZLB004	GCA_003184305.1	Animal	-	*estC*
Levilactobacillus hammesii DSM 16381	GCA_001434395.1	Sourdough	-	*estC*
Levilactobacillus koreensis 2625	GCA_001050435.1	Kimchi	-	*estC*
Levilactobacillus namurensis DSM 19117	GCA_001434785.1	Sourdough	-	-
Levilactobacillus namurensis NBRC 107158	GCA_007992015.1	Sourdough	-	-
Levilactobacillus parabrevis ATCC 53295	GCA_000383435.1	Other foods	-	-
Levilactobacillus parabrevis LMG 11984	GCA_001437515.1	NA	-	-
Levilactobacillus senmaizukei DSM 21775	GCA_001592085.1	NA	-	*estC*
Levilactobacillus spicheri DSM 15429	GCA_001435095.1	Sourdough	-	-
Levilactobacillus spicheri NBRC 107155	GCA_007991955.1	Sourdough	-	-
Levilactobacillus zymae ACA-DC 3411	GCA_900183405.1	Sourdough	-	*estC*
Levilactobacillus zymae DSM 19395	GCA_001434115.1	Sourdough	-	*estC*
Levilactobacillus zymae LZ395	GCA_009295845.1	Sourdough	-	*estC*
Levilactobacillus zymae NBRC 107157	GCA_007991995.1	Sourdough	-	*estC*
Ligilactobacillus agilis La3	GCA_002240375.2	Animal	-	-
Ligilactobacillus salivarius 2D	GCA_013487885.1	Animal	-	*estA*
Ligilactobacillus salivarius CECT 5317	GCA_000143435.1	Human	-	*estA*
Ligilactobacillus salivarius DJsa01	GCA_003316955.1	Animal	-	*estA*
Ligilactobacillus salivarius JCM 1046	GCA_000758365.1	Animal	-	*estA*
Ligilactobacillus salivarius Ren	GCA_001011095.1	Human	-	*estA*
Ligilactobacillus salivarius ZLS006	GCA_002162055.1	Animal	-	*estA*
Limosilactobacillus fermentum 222	GCA_001368755.1	Cocoa	-	*estC*
Limosilactobacillus fermentum 2760	GCA_008802915.1	Fermented dairy product	-	*estC*
Limosilactobacillus fermentum 3872	GCA_000466785.3	Human	-	*estC*
Limosilactobacillus fermentum AGR1485	GCA_011032765.1	Human	-	*estC*
Limosilactobacillus fermentum AGR1487	GCA_011032745.1	Human	-	*estC*
Limosilactobacillus fermentum CBA7106	GCA_003255875.1	Human	-	*estC*
Limosilactobacillus fermentum DR9	GCA_003710225.1	Fermented dairy product	-	*estC*
Limosilactobacillus fermentum F6	GCA_000397165.1	NA	-	*estC*
Limosilactobacillus fermentum FTDC8312	GCA_002119645.1	Human	-	*estC*
Limosilactobacillus fermentum HFD1	GCA_012273035.1	Human	-	*estC*
Limosilactobacillus fermentum IFO3956	GCA_000010145.1	NA	-	*estC*
Limosilactobacillus fermentum IMDO 130101	GCA_900205745.1	Sourdough	-	*estC*
Limosilactobacillus fermentum LACFRN 92	GCA_002192435.1	Human	-	*estC*
Limosilactobacillus fermentum LDTM 7301	GCA_003346795.1	Fermented drink	-	*estC*
Limosilactobacillus fermentum LMT275	GCA_003855655.1	Kimchi	-	*estC*

Limosilactobacillus fermentum MTCC 25067	GCA_002356135.1	Fermented dairy product	-	*estC*
Limosilactobacillus fermentum NCC 2970	GCA_001742205.1	NA	-	*estC*
Limosilactobacillus fermentum SK152	GCA_002242615.1	Fermented vegetable	-	*estC*
Limosilactobacillus fermentum SRCM 103285	GCA_004063515.1	Other foods	-	*estC*
Limosilactobacillus fermentum SRCM 103290	GCA_004063635.1	Other foods	-	*estC*
Limosilactobacillus fermentum USM 8633	GCA_009676625.1	Meat	-	*estC*
Limosilactobacillus fermentum YL11	GCA_003860425.1	Fermented dairy product	-	*estC*
Limosilactobacillus frumenti DSM 13145	GCA_001436045.1	Sourdough	-	-
Limosilactobacillus frumenti LF145	GCA_008876665.1	Sourdough	-	-
Limosilactobacillus mucosae A1	GCA_013423865.1	Human	-	*estC*
Limosilactobacillus panis DSM 6035	GCA_001435935.1	Sourdough	-	-
Limosilactobacillus pontis DSM 8475	GCA_001435345.1	Sourdough	-	-
Limosilactobacillus pontis LP475	GCA_009428965.1	Sourdough	-	-
Limosilactobacillus reuteri 1B	GCA_013487925.1	Animal	-	*estC*
Limosilactobacillus reuteri AN417	GCA_013348825.1	Animal	-	*estC*
Limosilactobacillus reuteri ATCC 53608	GCA_000236455.2	Human	-	*estC*
Limosilactobacillus reuteri ATGF4	GCA_004208615.1	Human	-	*estC*
Limosilactobacillus reuteri Byunre01	GCA_003316895.1	Animal	-	*estC*
Limosilactobacillus reuteri CNEIKCA3	GCA_013694365.1	Animal	-	*estC*
Limosilactobacillus reuteri DSM 20016	GCA_000016825.1	Human	-	*estC*
Limosilactobacillus reuteri I49	GCA_001688685.2	Animal	-	*estC*
Limosilactobacillus reuteri I5007	GCA_000410995.1	Animal	-	*estC*
Limosilactobacillus reuteri IRT	GCA_001046835.1	NA	-	*estC*
Limosilactobacillus reuteri JCM 1112	GCA_000010005.1	Human	-	*estC*
Limosilactobacillus reuteri LL7	GCA_007633215.1	Animal	-	*estC*
Limosilactobacillus reuteri reuteri	GCA_009184725.1	Human	-	*estC*
Limosilactobacillus reuteri SD2112	GCA_000159455.2	Human	-	*estC*
Limosilactobacillus reuteri SKKUOGDONS01	GCA_003316935.1	Animal	-	*estC*
Limosilactobacillus reuteri TD1	GCA_000439275.1	NA	-	*estC*
Limosilactobacillus reuteri WHH1689	GCA_003072625.1	Wine	-	*estC*
Limosilactobacillus reuteri YSJL12	GCA_006874665.1	Animal	-	*estC*
Limosilactobacillus reuteri ZLR003	GCA_001618905.1	Animal	-	*estC*
Limosilactobacillus secaliphilus DSM 17896	GCA_001437055.1	Sourdough	-	*estC*
Limosilactobacillus vaginalis LV515	GCA_009362935.1	Human	-	*estA*, *estC*
Liquorilactobacillus uvarum DSM 19971	GCA_001434935.1	Other foods	*estB*	*estB*
Loigolactobacillus coryniformis CBA3616	GCA_007954685.1	Kimchi	-	*estC*
Loigolactobacillus coryniformis DSM 20001	GCA_002706425.1	Environment	-	-
Loigolactobacillus coryniformis DSM 20004	GCA_002706705.1	Animal	-	-
Schleiferilactobacillus harbinensis M1	GCA_009217765.1	Other foods	*estA*	*estA*, *estC*
Schleiferilactobacillus harbinensis NSMJ42	GCA_008694105.1	Fermented drink	*estA*	*estA*, *estC*

Schleiferilactobacillus perolens DSM 12744	GCA_001435585.1	Other foods	*estA*	*estA*, *estC*
Secundilactobacillus malefermentans CBA3618	GCA_008033155.1	Kimchi	*estC*	*estC*
Secundilactobacillus odoratitofui DSM 19909	GCA_001313185.1	NA	-	*estA*
Secundilactobacillus ollinoides JCM 1123	GCA_001312845.1	NA	*estA*	*estC*

Table S3. Yeast strains screened by PCR assays developed during the present study to target either acetate ester (*ATF1*/*ATF2*) or ethyl ester (*EHT1*/*EBB1*) biosynthesis genes and the presence (+) or absence (-) of amplification. All strains were stored at the culture collection of the research group of Industrial Microbiology and Food Biotechnology (IMDO-VUB), unless stated otherwise.

Strain	Isolation source	Acetate ester genes	Ethyl ester genes
Dekkera anomala G-B-WL-Y5	Belgian bakery sourdough	-	-
Dekkera anomala G-B-W-Y8	Belgian laboratory sourdough	-	-
Dekkera anomala G-B-W-Y	Belgian laboratory sourdough	-	-
Dekkera anomala G-B-WL-Y13	Belgian laboratory sourdough	-	+
Dekkera bruxellensis BLl-Yc2	Belgian laboratory sourdough	-	-
Dekkera bruxellensis BLl-Yc24	Belgian laboratory sourdough	-	-
Maudiozymabarnettii MUCL 51212	Belgian bakery sourdough	-	-
Maudiozymabulderi E-B-S-Y1	Belgian bakery sourdough	-	+
Maudiozymabulderi E-B-R-Y1	Belgian bakery sourdough	-	+
Maudiozymabulderi E-B-R-Y2	Belgian bakery sourdough	+	-
Maudiozymabulderi E-B-W-Y24	Belgian bakery sourdough	+	+
Maudiozymabulderi E-B-M-Y24	Belgian bakery sourdough	-	-
Maudiozymahumilis F-NY-R-Y1	USA bakery sourdough	-	-
Maudiozymahumilis F-NY-WR-Y5	USA bakery sourdough	+	-
Maudiozymahumilis F-NY-WW-Y3	USA bakery sourdough	-	-
Monosporozymaunispora LC400-Mal2-Y1	Belgian laboratory sourdough	-	-
Monosporozymaunispora LC400-Mal1-Y27	Belgian laboratory sourdough	-	-
Monosporozymaunispora B-B-R-Y1	Belgian bakery sourdough	-	-
Monosporozymaunispora MUCL 51234	Belgian bakery sourdough	-	-
Nakaseomyces glabratus LC200-Aj1-Y11	Belgian laboratory sourdough	-	-
Naumovozyma castellii BLw-Yc1	Belgian laboratory sourdough	-	-
Naumovozyma castellii BLw-Yc9	Belgian laboratory sourdough	-	-
Naumovozyma castellii BLw-Yc21	Belgian laboratory sourdough	-	-
Naumovozyma castellii BLw-Y23	Belgian laboratory sourdough	-	-
Pichia cecembensis LC200-Lj1-Y21	Belgian laboratory sourdough	-	-
Pichia cecembensis BLw-Y24	Belgian laboratory sourdough	-	-
Pichia fermentans C-B-R-Y1	Belgian bakery sourdough	-	+
Pichia fermentans C-B-R-Y8	Belgian bakery sourdough	-	+
Pichia kudriavzevii LC400-Mal2-Y27	Belgian laboratory sourdough	-	-
Pichia kudriavzevii LC400-Mal2-Y27	Belgian laboratory sourdough	-	-
Saccharomyces cerevisiae LC400-Ctl1-Y30	Belgian laboratory sourdough	+	+
Saccharomyces cerevisiae A-UK-R-Y6	UK bakery sourdough	+	+
Saccharomyces cerevisiae A-UK-R-Y7	UK bakery sourdough	+	+
Saccharomyces cerevisiae D-F-K-Y1	French bakery sourdough	+	+
Saccharomyces cerevisiae D-F-K-Y24	French bakery sourdough	+	+

Saccharomyces cerevisiae D-F-S-Y1	French bakery sourdough	+	-
Saccharomyces cerevisiae D-F-S-Y24	French bakery sourdough	+	+
Saccharomyces cerevisiae D-F-B-Y1	French bakery sourdough	+	-
Saccharomyces cerevisiae D-F-R-Y1	French bakery sourdough	+	+
Saccharomyces cerevisiae D-F-W-Y1	French bakery sourdough	+	+
Saccharomyces cerevisiae D-F-W-Y24	French bakery sourdough	+	+
Saccharomyces cerevisiae LC200-Lj3-Y21	Belgian laboratory sourdough	+	-
Saccharomyces cerevisiae LC200-BLjw-Y74	Belgian laboratory sourdough	+	-
Saccharomyces cerevisiae E-B-S-Y8	Belgian bakery sourdough	+	-
Saccharomyces cerevisiae E-B-W-Y17	Belgian bakery sourdough	+	+
Saccharomyces cerevisiae BLl-Y18	Belgian laboratory sourdough	+	+
Saccharomyces cerevisiae C-B-R-Y16	Belgian bakery sourdough	+	+
Saccharomyces cerevisiae C-B-R-Y17	Belgian bakery sourdough	+	-
Saccharomyces cerevisiae MUCL 51208	Belgian bakery sourdough	+	-
Saccharomyces cerevisiae MUCL 51209	Belgian bakery sourdough	+	+
Saccharomyces cerevisiae MUCL 51210	Belgian bakery sourdough	+	+
Saccharomyces cerevisiae MUCL 51213	Belgian bakery sourdough	+	+
Saccharomyces cerevisiae MUCL 51214	Belgian bakery sourdough	+	+
Saccharomyces cerevisiae MUCL 51215	Belgian bakery sourdough	+	+
Saccharomyces cerevisiae MUCL 51216	Belgian bakery sourdough	+	+
Saccharomyces cerevisiae MUCL 51217	Belgian bakery sourdough	+	+
Saccharomyces cerevisiae MUCL 51219	Belgian bakery sourdough	+	+
Saccharomyces cerevisiae MUCL 51221	Belgian bakery sourdough	+	+
Saccharomyces cerevisiae MUCL 51223	Belgian bakery sourdough	+	+
Saccharomyces cerevisiae MUCL 51226	Belgian bakery sourdough	+	+
Saccharomyces cerevisiae MUCL 51227	Belgian bakery sourdough	+	+
Saccharomyces cerevisiae MUCL 51230	Belgian bakery sourdough	+	+
Saccharomyces cerevisiae MUCL 51232	Belgian bakery sourdough	-	+
Saccharomyces cerevisiae MUCL 51233	Belgian bakery sourdough	-	+
Saccharomyces cerevisiae MUCL 51235	Belgian bakery sourdough	-	+
Saccharomyces cerevisiae MUCL 51236	Belgian bakery sourdough	-	+
Saccharomyces paradoxus LC200-BLjj-Y61	Belgian laboratory sourdough	-	+
Saccharomyces paradoxus LC200-BLjj-Y100	Belgian laboratory sourdough	-	+
Saccharomyces paradoxus BLl-Y1	Belgian laboratory sourdough	-	+
Saccharomyces paradoxus LC200-BLjw-Y51	Belgian laboratory sourdough	-	+
Torulaspora delbrueckii LC400-Mal1-Y15	Belgian laboratory sourdough	-	-
Torulaspora delbrueckii MUCL 51218	Belgian bakery sourdough	-	-
Torulaspora delbrueckii MUCL 51211	Belgian bakery sourdough	-	-
Wickerhamomyces anomalus Lbcru+citbuff	Belgian laboratory sourdough	-	+
Wickerhamomyces anomalus cruB_20150309-Y10	Belgian laboratory sourdough	-	+

Wickerhamomyces anomalus LC400-Ctl2-Y1	Belgian laboratory sourdough	-	+
Wickerhamomyces anomalus LC400-Mal1-Y24	Belgian laboratory sourdough	-	+
Wickerhamomyces anomalus LC400-Mal2-Y26	Belgian laboratory sourdough	-	+
Wickerhamomyces anomalus LC200-Lj3-Y22	Belgian laboratory sourdough	-	+
Wickerhamomyces anomalus LC200-Aj2-Y11	Belgian laboratory sourdough	-	+
Wickerhamomyces anomalus LC200-Aj1-Y14	Belgian laboratory sourdough	-	+
Wickerhamomyces anomalus LC200-Aj3-Y11	Belgian laboratory sourdough	-	+
Wickerhamomyces anomalus MUCL 51207	Belgian bakery sourdough	-	+
Wickerhamomyces anomalus MUCL 51220	Belgian bakery sourdough	-	+
Wickerhamomyces anomalus MUCL 51222	Belgian bakery sourdough	-	+
Wickerhamomyces anomalus MUCL 51224	Belgian bakery sourdough	-	+
Wickerhamomyces anomalus MUCL 51225	Belgian bakery sourdough	-	-
Wickerhamomyces anomalus MUCL 51228	Belgian bakery sourdough	-	+
Wickerhamomyces anomalus MUCL 51229	Belgian bakery sourdough	-	+
Wickerhamomyces anomalus MUCL 51231	Belgian bakery sourdough	-	+
Wickerhamomyces anomalus MUCL 51237	Belgian bakery sourdough	-	+

Table S4. Accession numbers of the yeast protein sequences used for the primer design during the development of the PCR assays to target either acetate ester or ethyl ester biosynthesis genes.

Accession number	Yeast species
**ATF1/ATF2 (acetate esters)**	
XP_003956955	*Kazachstania africana*
XP_002551954, XP_002552712	*Lachancea thermotolerans*
XP_445666	*Nakaseomyces glabratus*
XP_003673631	*Naumovozyma castellii*
EJS41554.1	*Saccharomyces arboricola*
KOH50517.1, KOH47626.1	*Saccharomyces boulardii*
NP_011693.1, NP_015022.3	*Saccharomyces cerevisiae*
EHN06953.1, EHN00030.1	*Saccharomyces cerevisiae x Saccharomyces kudriavzevii*
XP_018222382.1, EJT43039.1, XP_018219565.1	*Saccharomyces eubayanus*
EJT43925.1	*Saccharomyces kudriavzevii*
XP_033766513.1, XP_033769481.1	*Saccharomyces paradoxus*
AAP72993.1, AAP72991.1	*Saccharomyces pastorianus*
AEZ66621.1, AEZ66622.1	*Wickerhamomyces anomalus*
**EHT1/EEB1 (ethyl esters)**	
EJS44777.1, EJS41448.1	*Saccharomyces arboricola*
NP_009736.3, Q02891	*Saccharomyces cerevisiae*
EHN03619.1, EHM99903.1	*Saccharomyces cerevisiae x Saccharomyces kudriavzevii*
XP_018223209.1, XP_018218938.1	*Saccharomyces eubayanus*
EJT44508.1, EJT43110.1	*Saccharomyces kudriavzevii*
XP_033764769.1, XP_033769672.1	*Saccharomyces paradoxus*
QID84351.1, QID82880.1	*Saccharomyces pastorianus*

Supplementary Figures

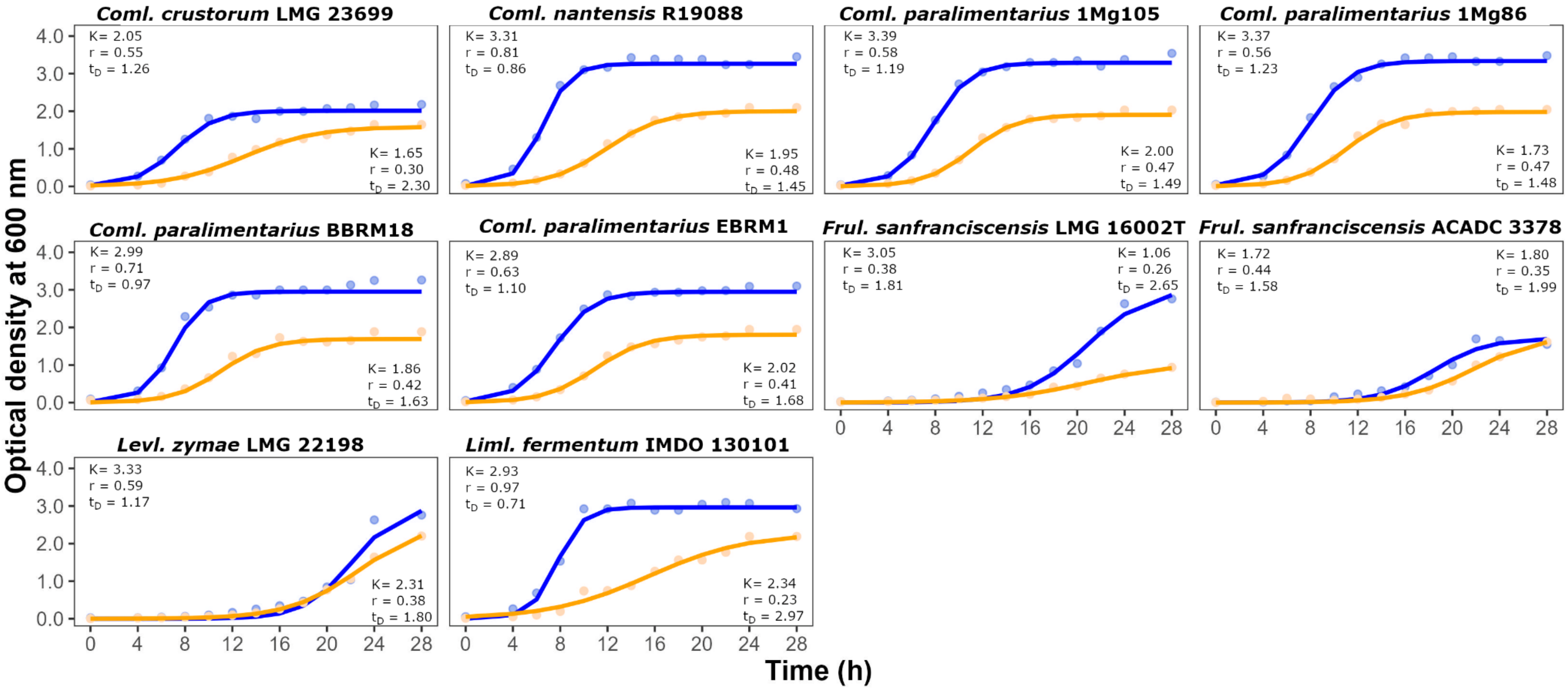

Figure S1. Growth course of selected lactic acid bacteria strains in a wheat sourdough simulation medium (WSSM) and a modified version of this medium containing ester precursor molecules (mWSSM). The dots represent the optical density measurements of fermentation processes performed in duplicate, and the lines represent the data modelled by the logistic equation. K, maximum population density; r, specific growth rate; t_D, doubling time.

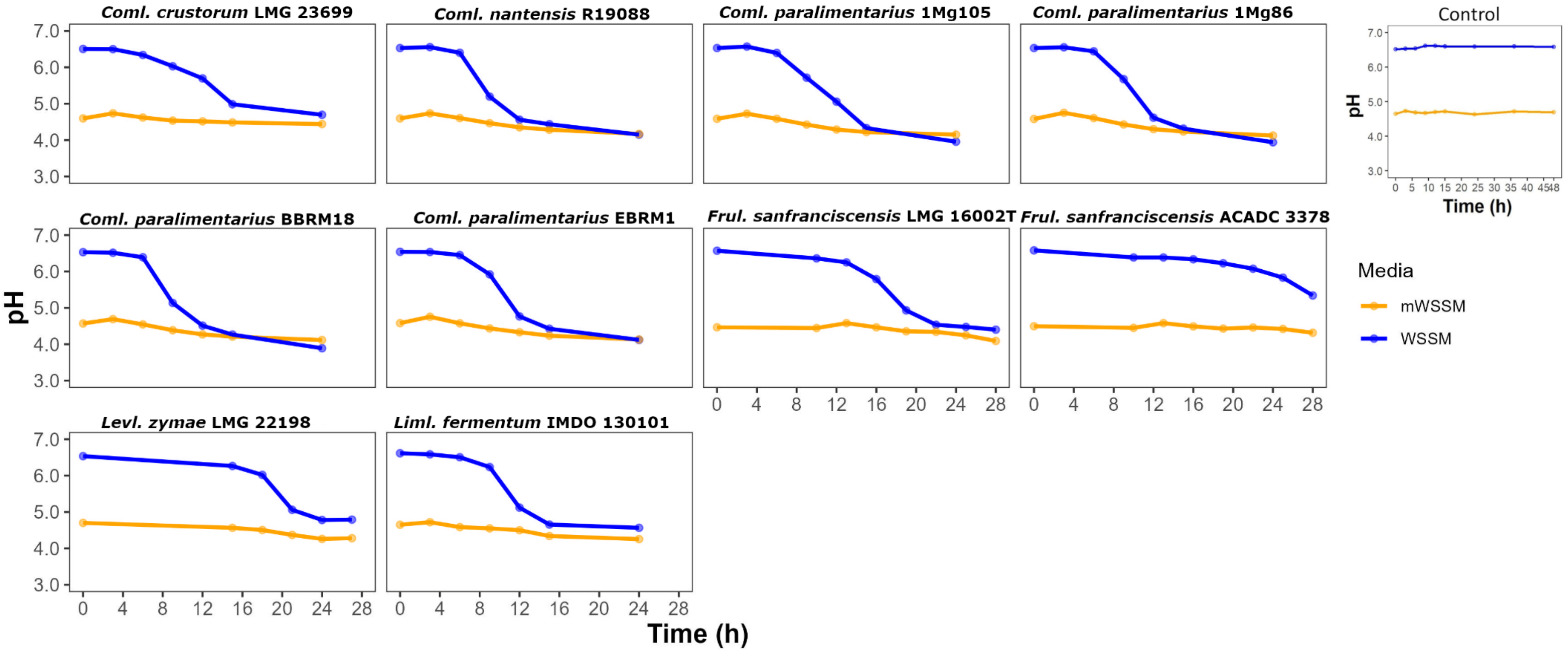

Figure S2. Course of the pH during fermentation processes carried out with selected lactic acid bacteria strains in a wheat sourdough simulation medium (WSSM) and a modified version of this medium containing ester precursor molecules (mWSSM). The control fermentation processes were carried out without the inoculation of any of the lactic acid bacteria strains. The symbols represent the pH measurements of fermentations processes perfomed in duplicate.
